# Supplementary material for: Enzymatic protein fusions with 100% product yield
Source: eLife. 2025 Apr 1;13:RP102765. doi: 10.7554/eLife.102765 (PMC11961121; doi:10.7554/eLife.102765)
Supplement: Figure 2—source data 1. [file elife-102765-fig2-data1.pdf]

Gel 1 (A)

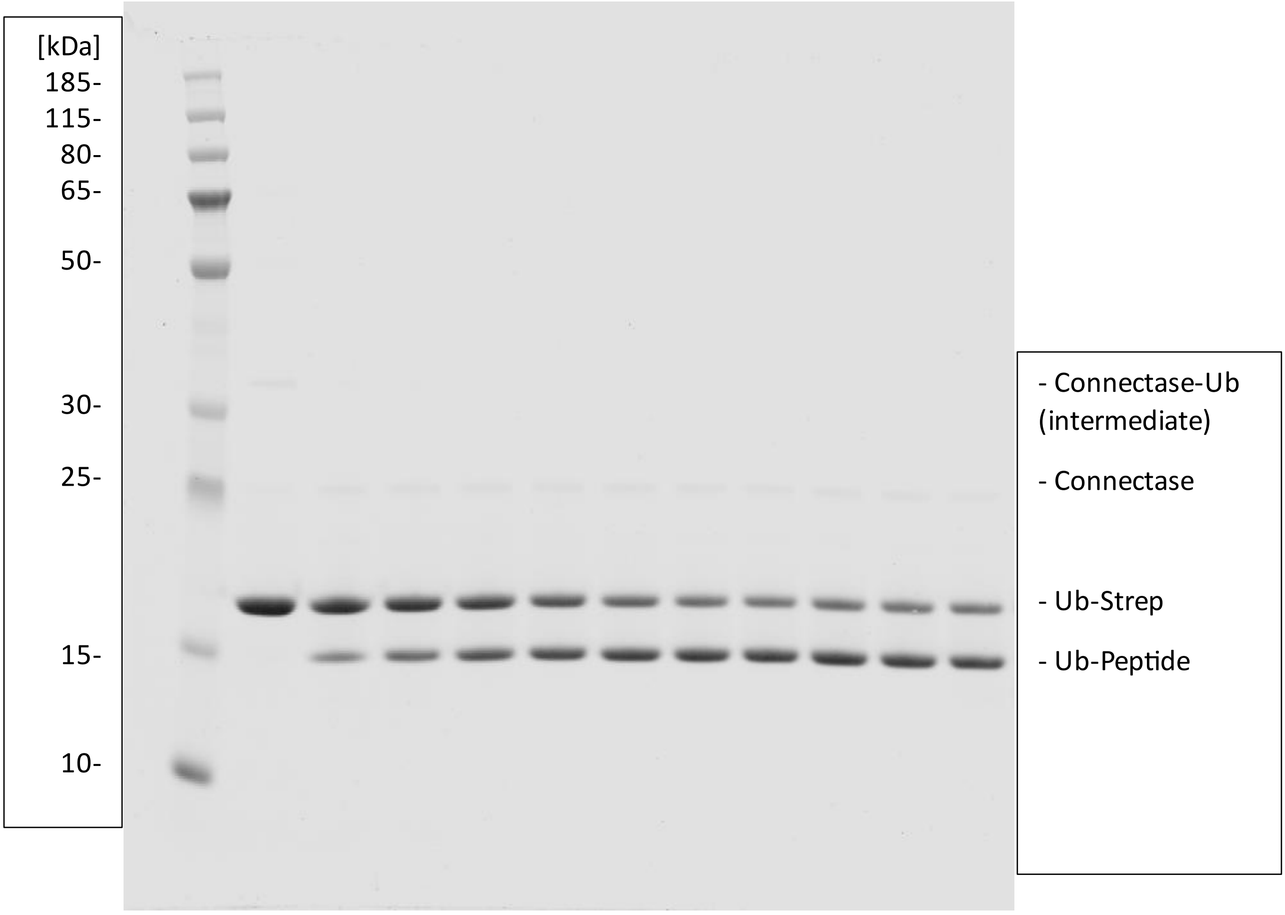

Lane	1	2	3	4	5	6	7	8	9	10	11
Marker	0	0.1	0.3	0.5	1	2	4	8	24	28	96
	Time [h]										

Gel 2 (C)

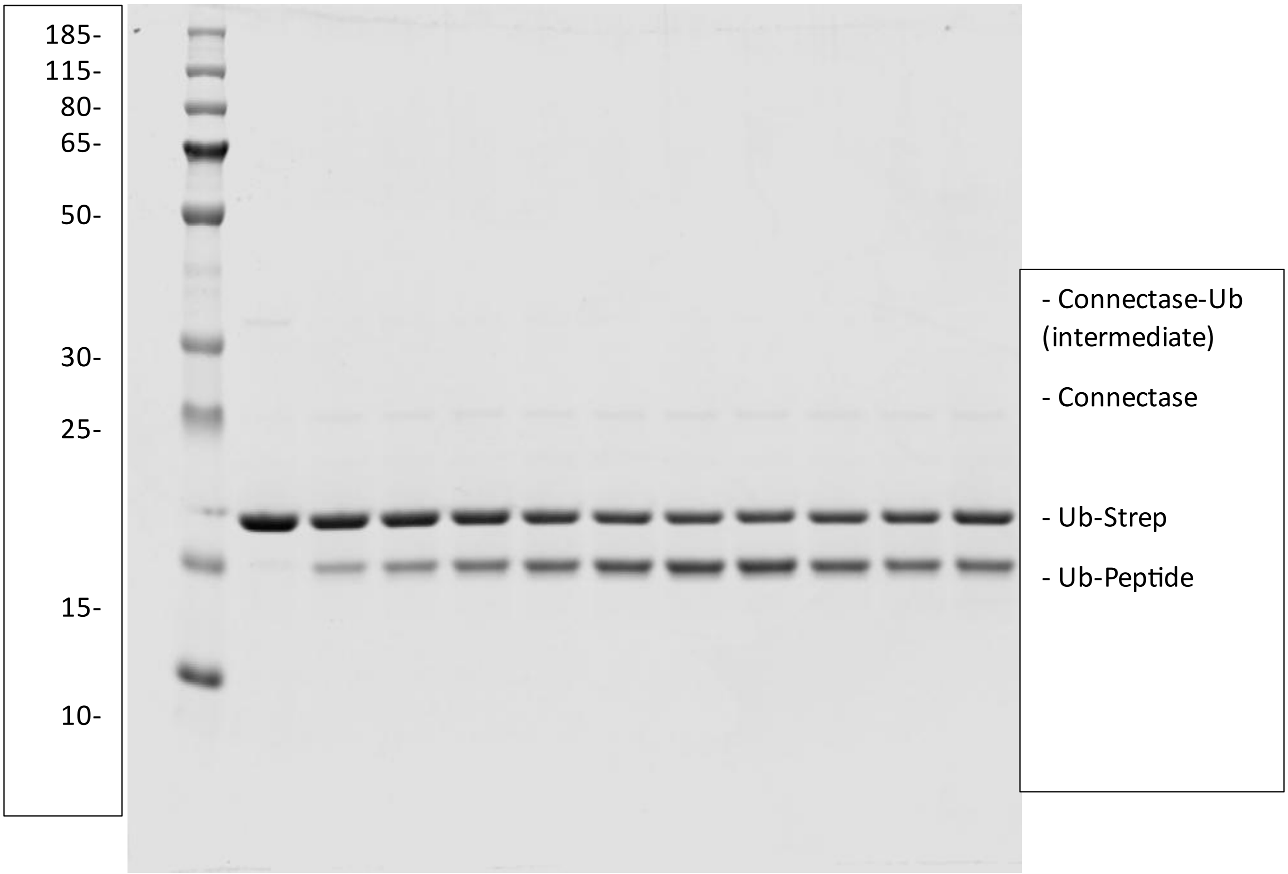

Lane	1	2	3	4	5	6	7	8	9	10	11
Marker	0	0.1	0.3	0.5	1	2	4	8	24	28	96
	Time [h]										

Gel 3 (D)

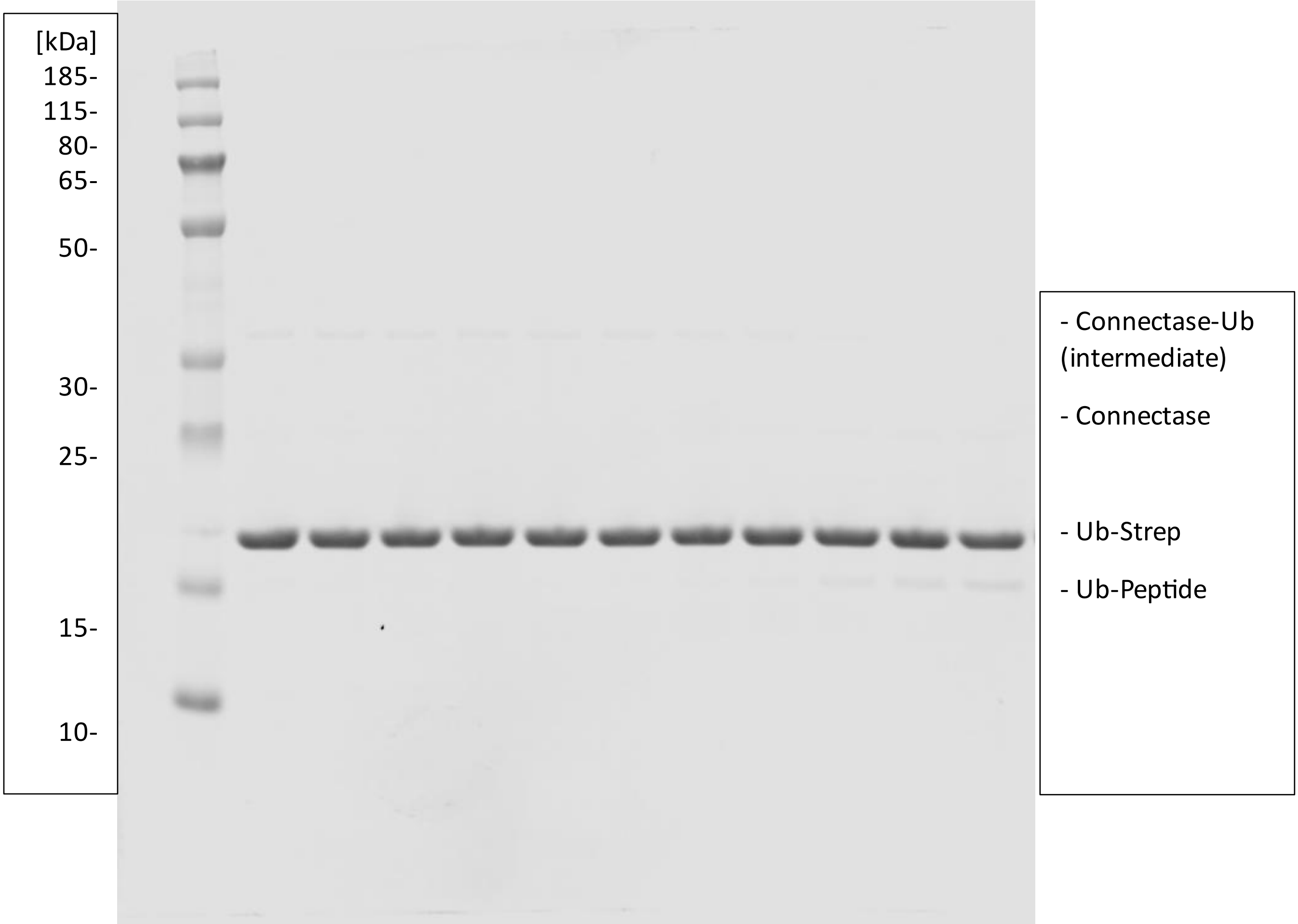

Lane	1	2	3	4	5	6	7	8	9	10	11
Marker	0	0.1	0.3	0.5	1	2	4	8	24	28	96
	Time [h]										

Gel 4 (E)

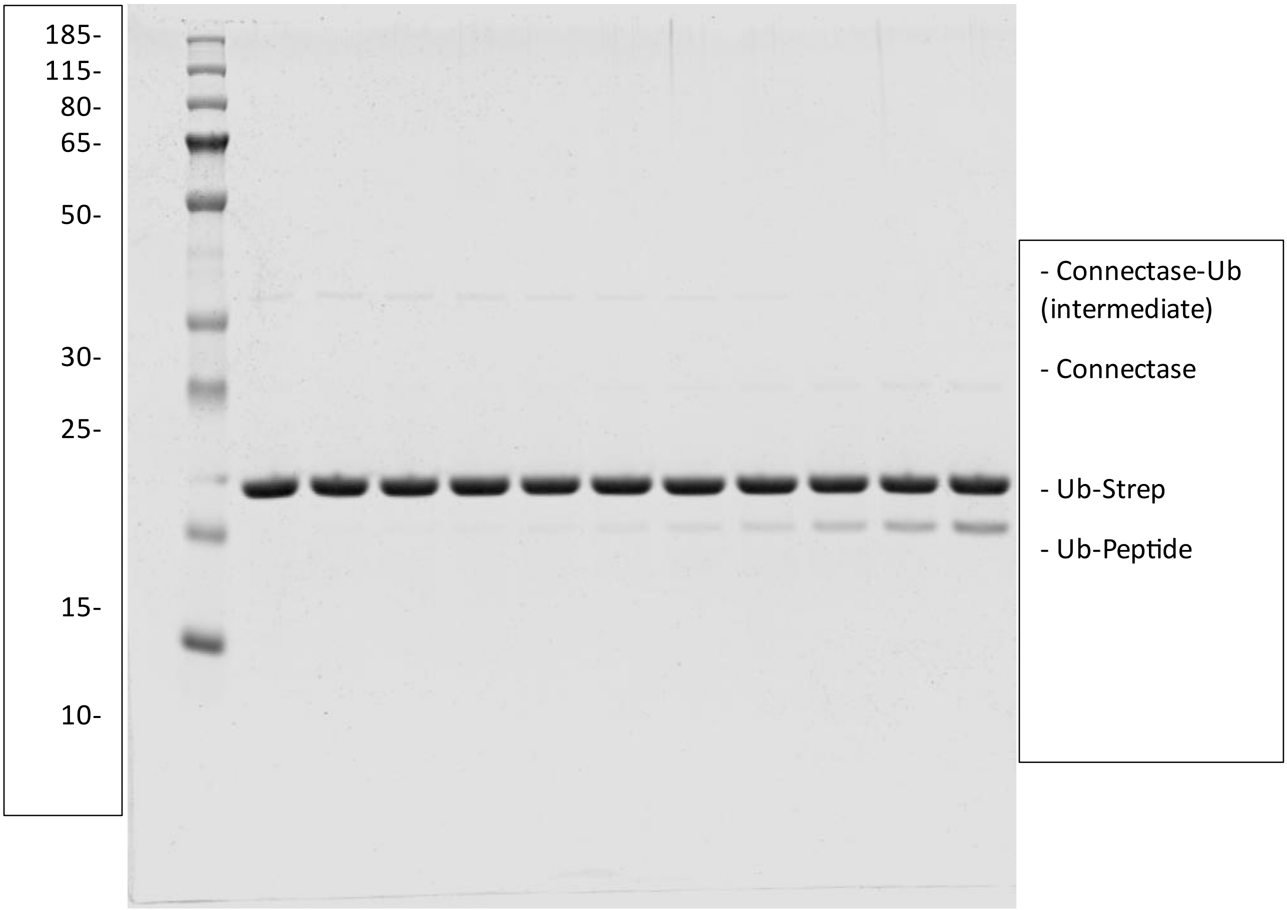

Lane	1	2	3	4	5	6	7	8	9	10	11
Marker	0	0.1	0.3	0.5	1	2	4	8	24	28	96
	Time [h]										

Gel 5 (F)

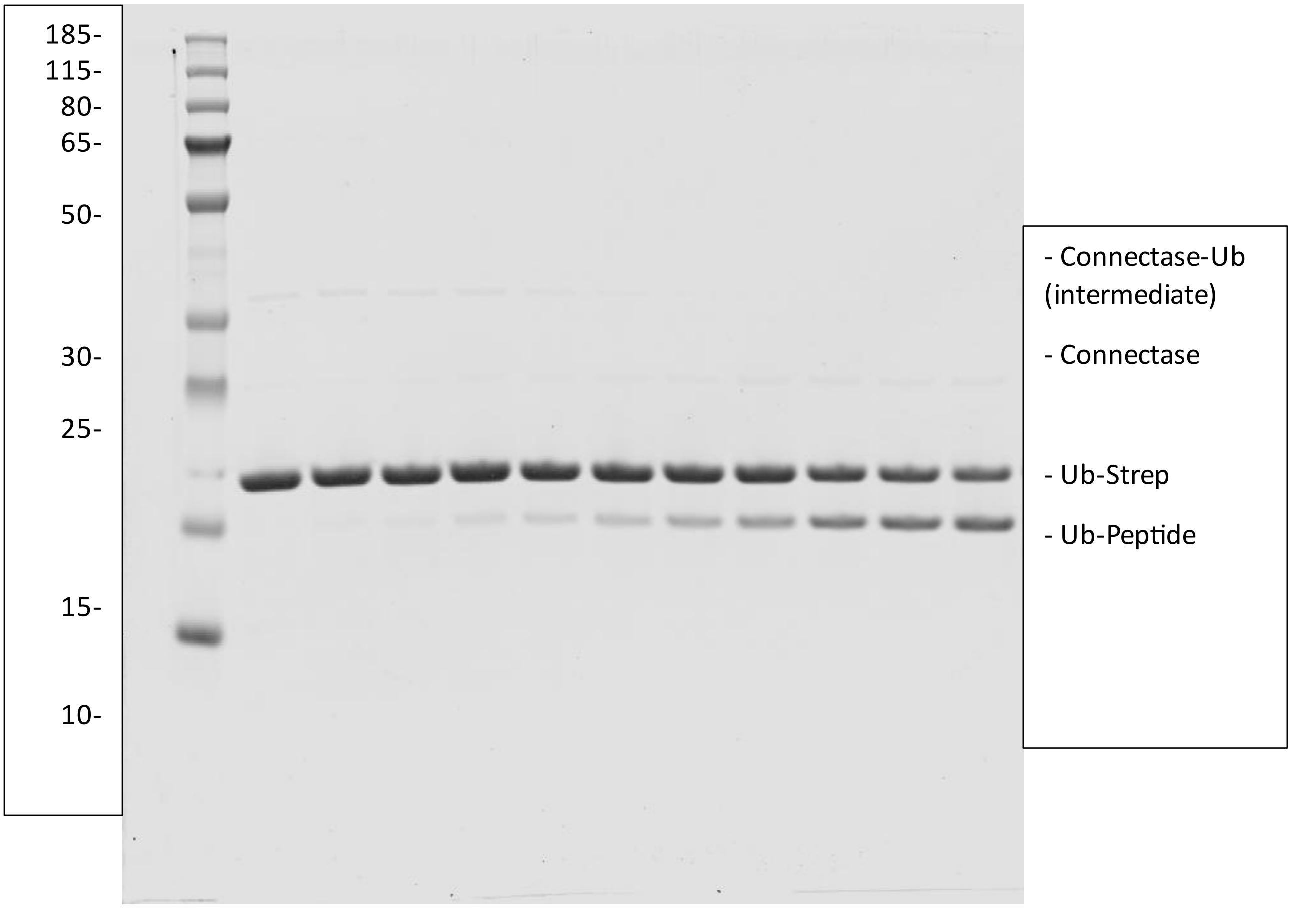

Lane	1	2	3	4	5	6	7	8	9	10	11
Marker	0	0.1	0.3	0.5	1	2	4	8	24	28	96
	Time [h]										

Gel 6 (G)

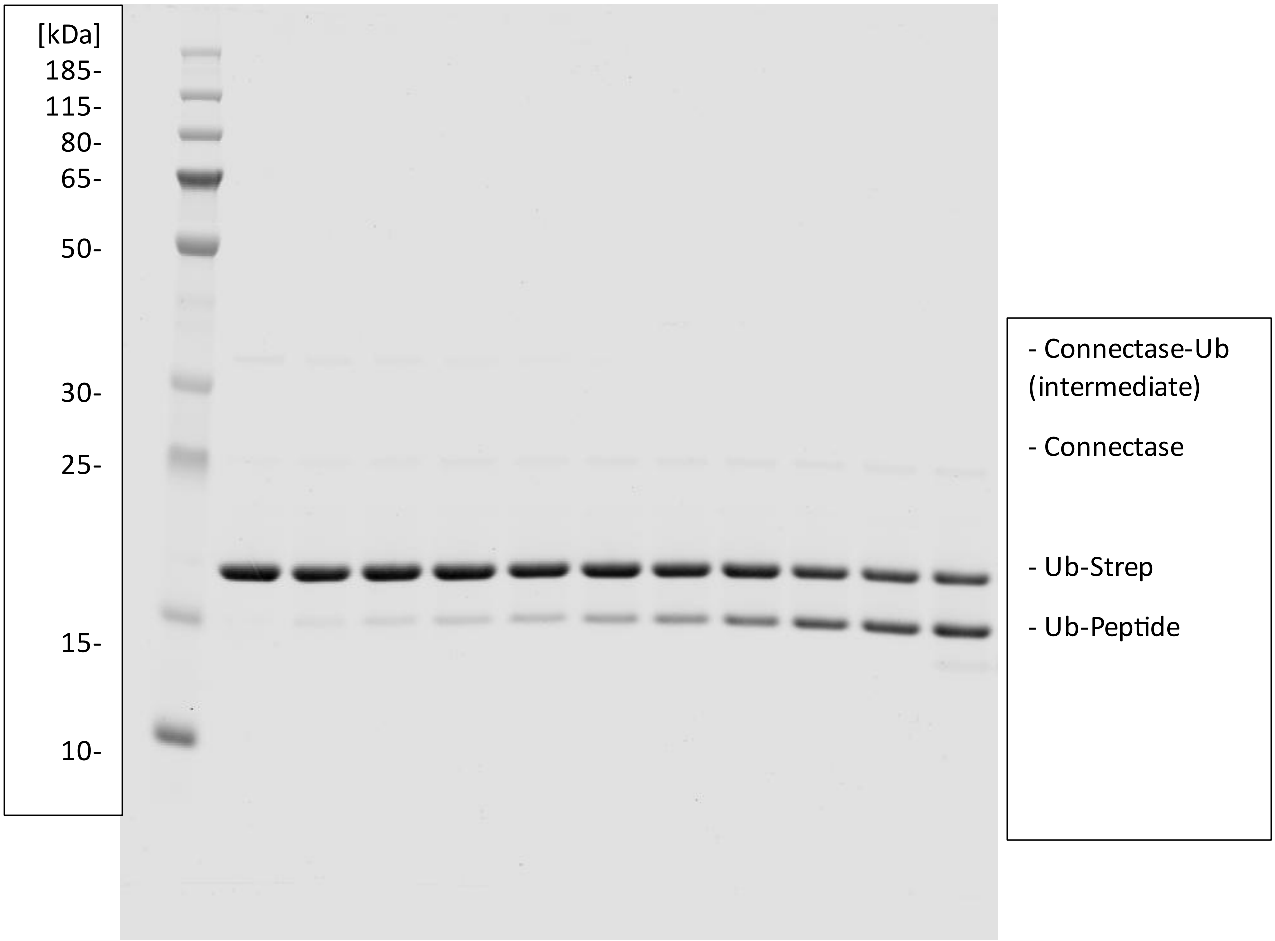

Lane	1	2	3	4	5	6	7	8	9	10	11
Marker	0	0.1	0.3	0.5	1	2	4	8	24	28	96
	Time [h]										

Gel 7 (H)

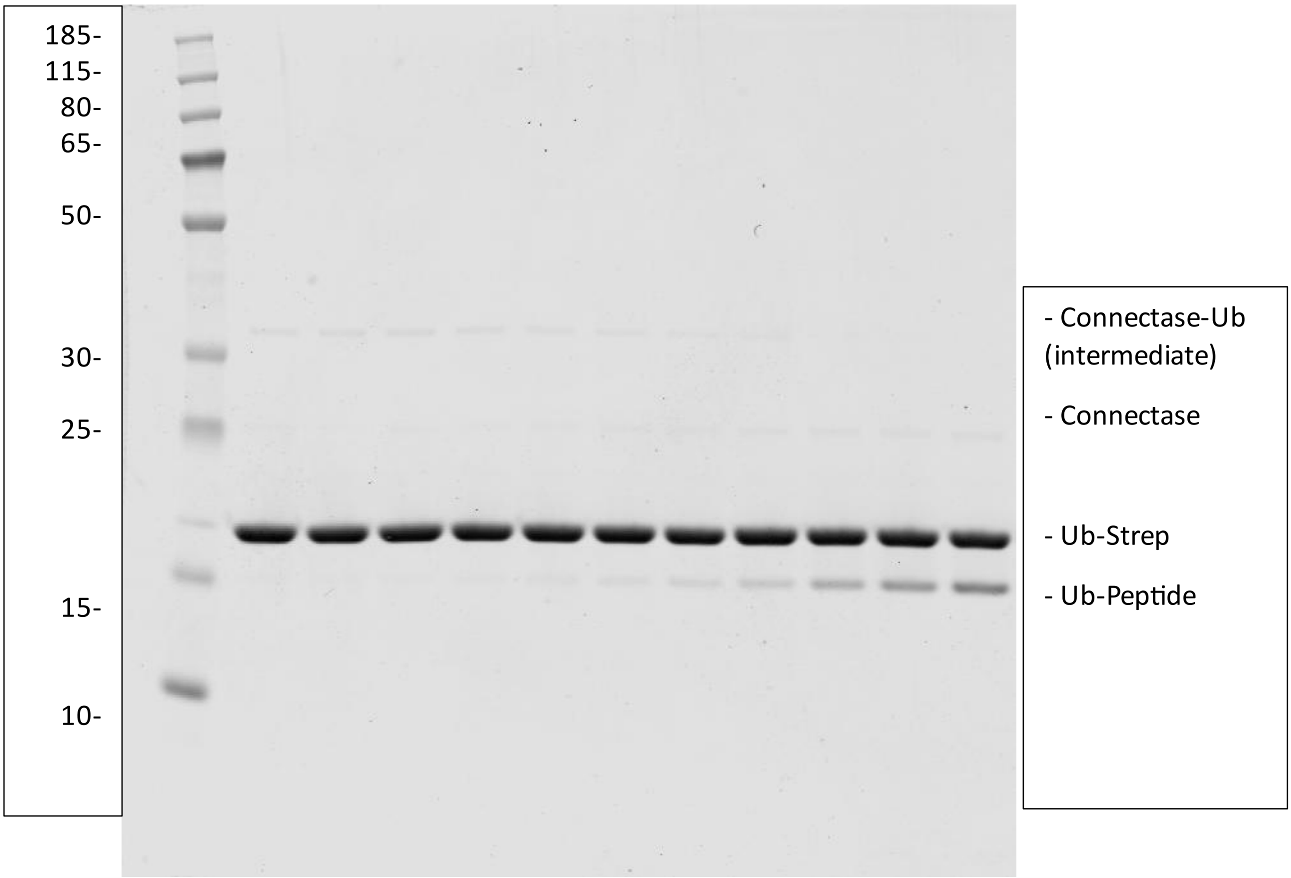

Lane	1	2	3	4	5	6	7	8	9	10	11
Marker	0	0.1	0.3	0.5	1	2	4	8	24	28	96
	Time [h]										

Gel 8 (I)

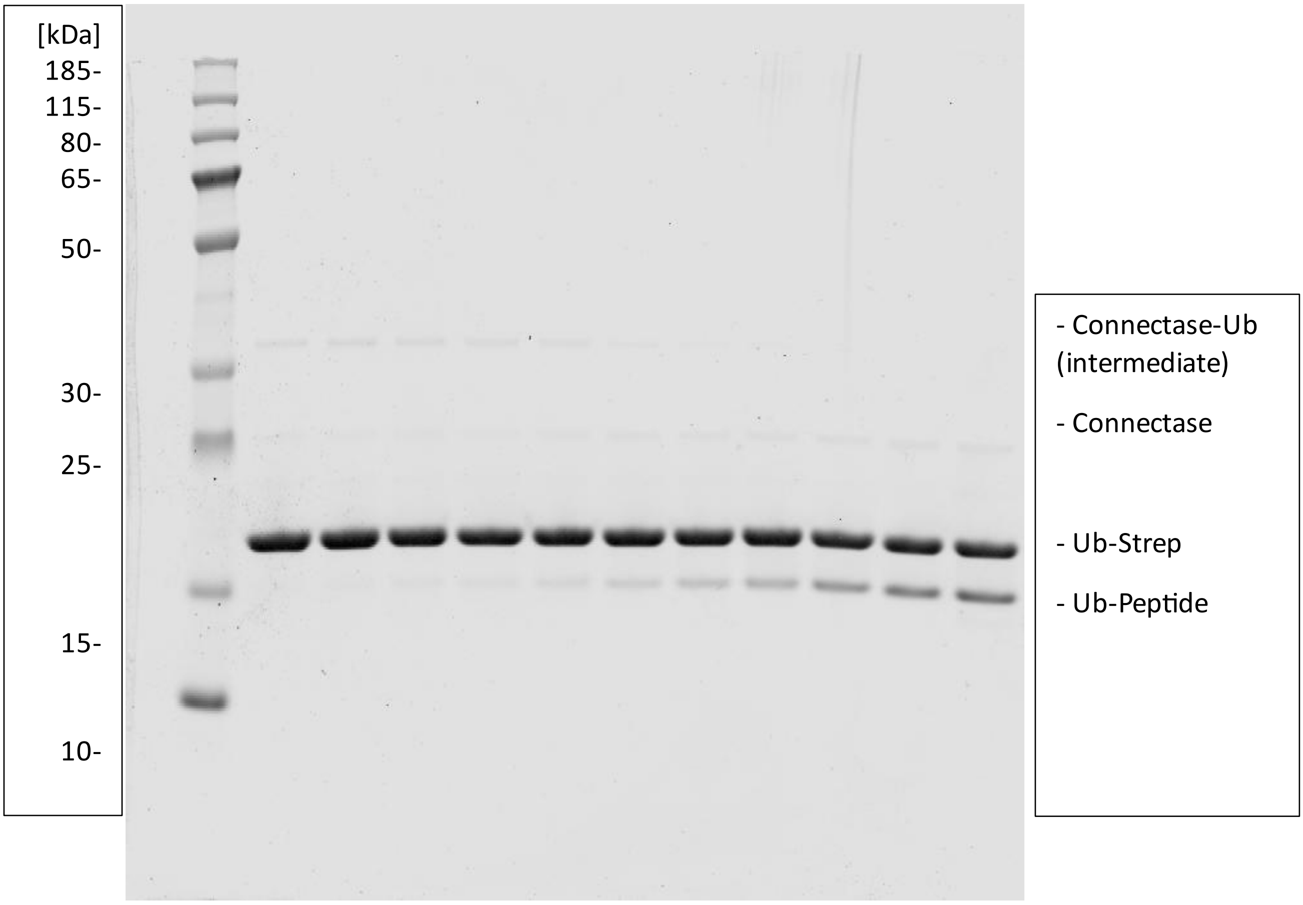

Lane	1	2	3	4	5	6	7	8	9	10	11
Marker	0	0.1	0.3	0.5	1	2	4	8	24	28	96
	Time [h]										

Gel 9 (K)

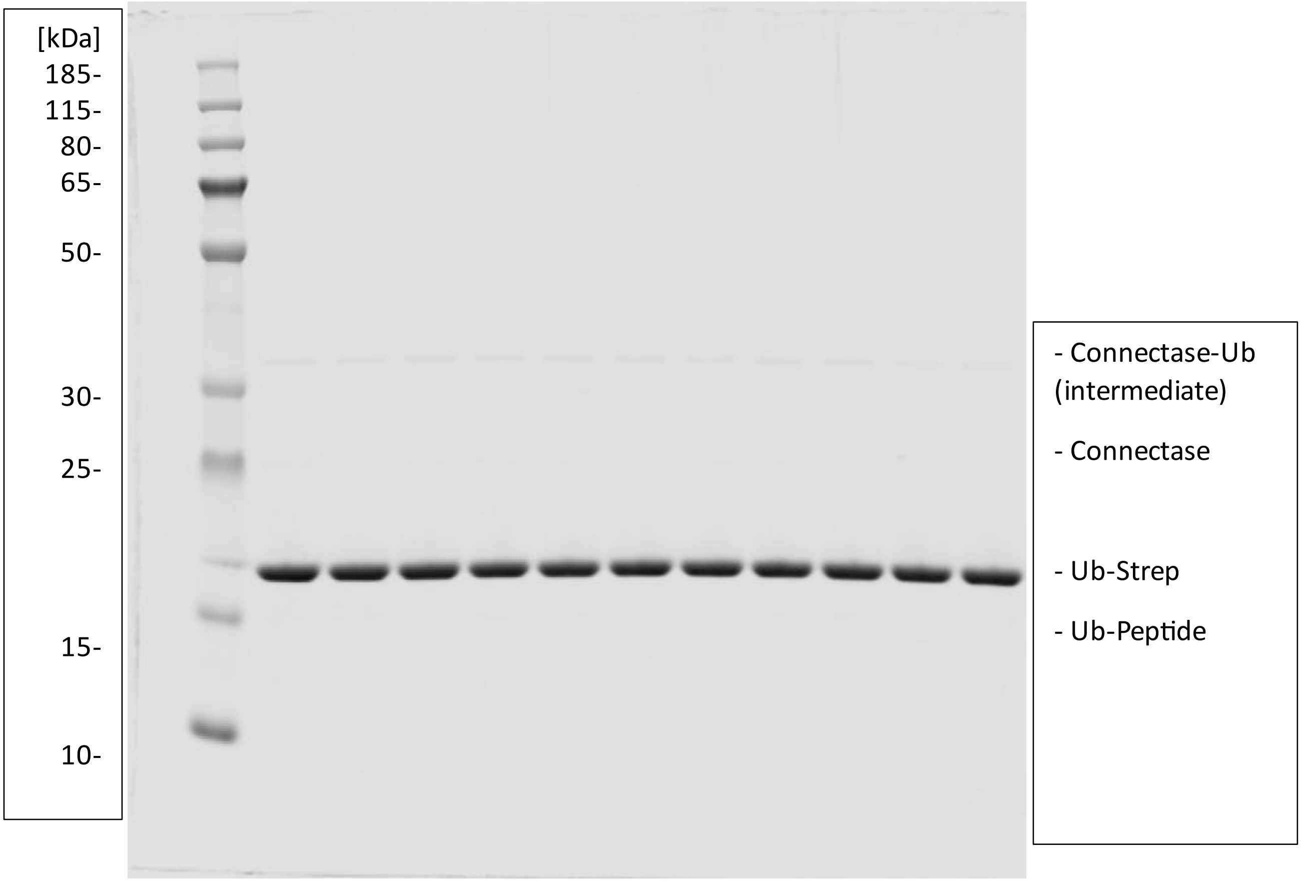

Lane	1	2	3	4	5	6	7	8	9	10	11
Marker	0	0.1	0.3	0.5	1	2	4	8	24	28	96
	Time [h]										

Gel 10 (L)

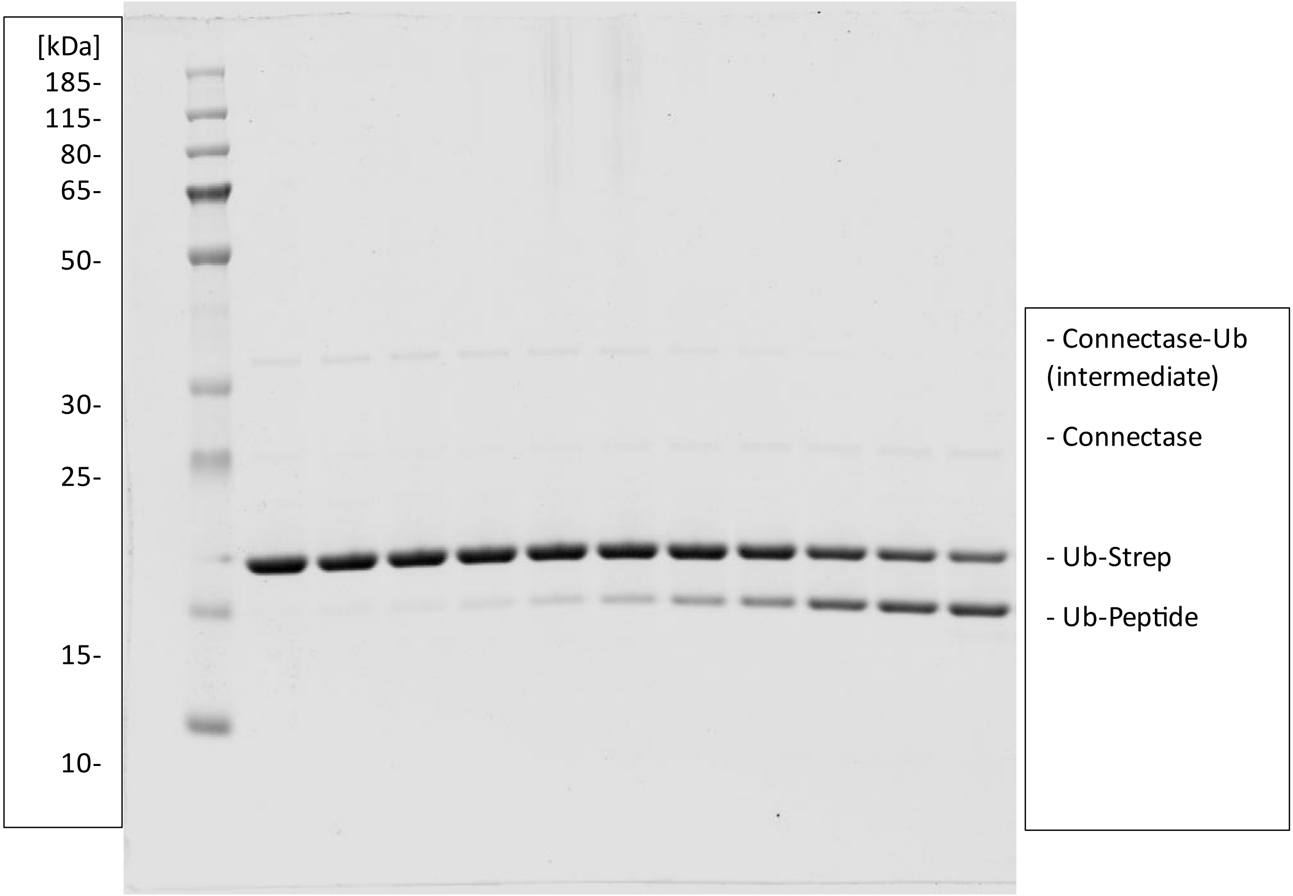

Lane	1	2	3	4	5	6	7	8	9	10	11
Marker	0	0.1	0.3	0.5	1	2	4	8	24	28	96
	Time [h]										

Gel 11 (M)

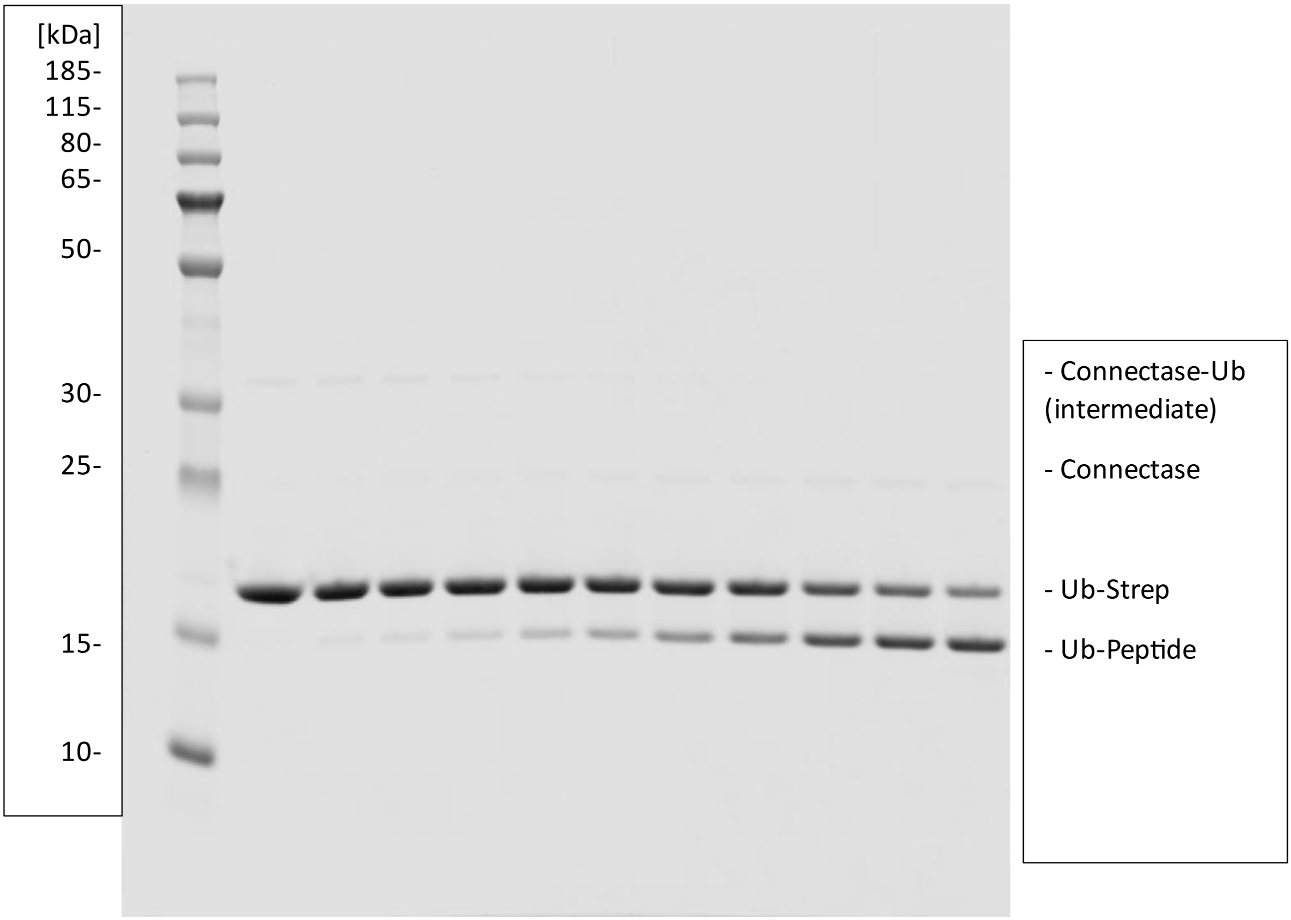

Lane	1	2	3	4	5	6	7	8	9	10	11
Marker	0	0.1	0.3	0.5	1	2	4	8	24	28	96
	Time [h]										

Gel 12 (N)

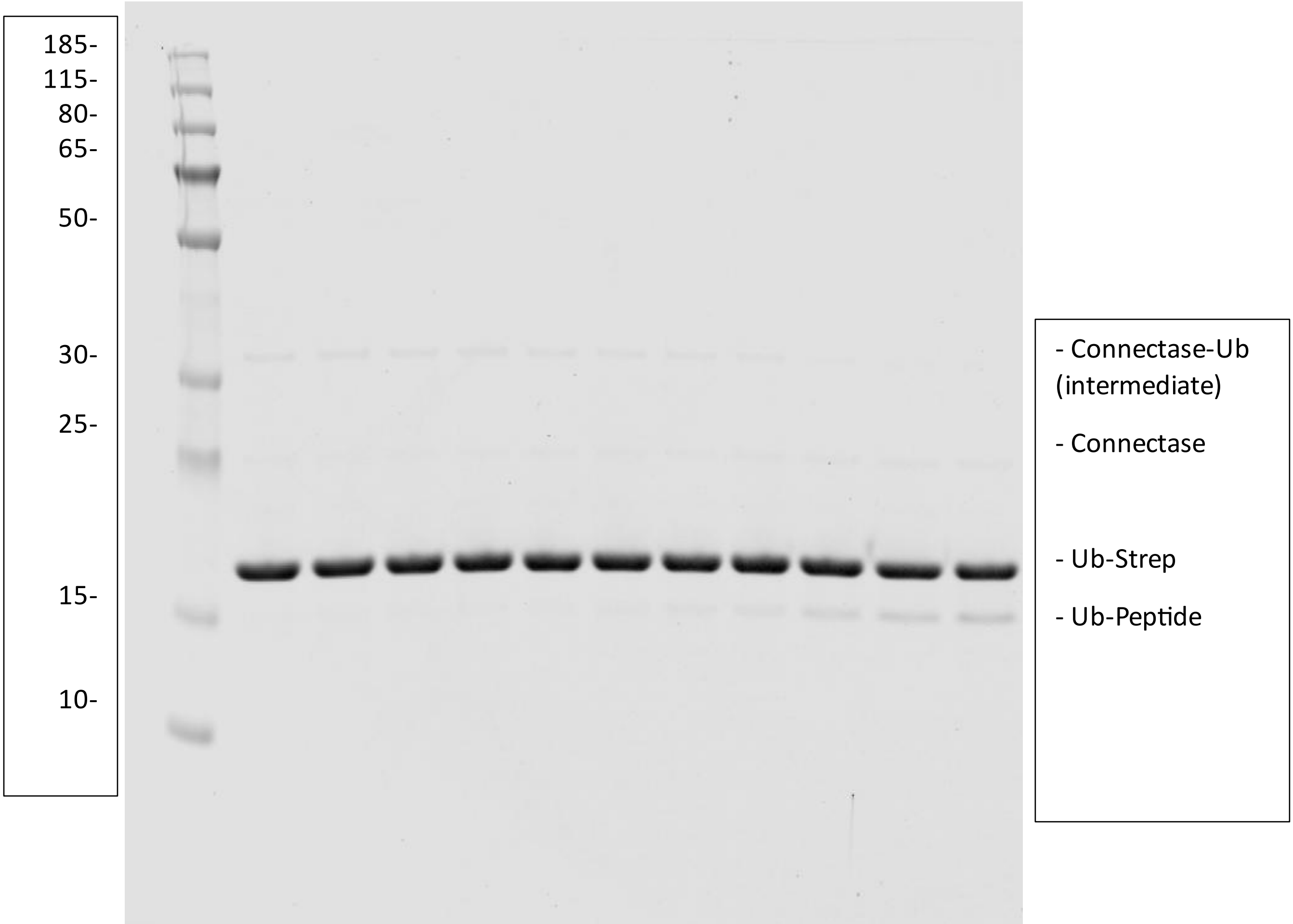

Lane	1	2	3	4	5	6	7	8	9	10	11
Marker	0	0.1	0.3	0.5	1	2	4	8	24	28	96
	Time [h]										

Gel 13 (P)

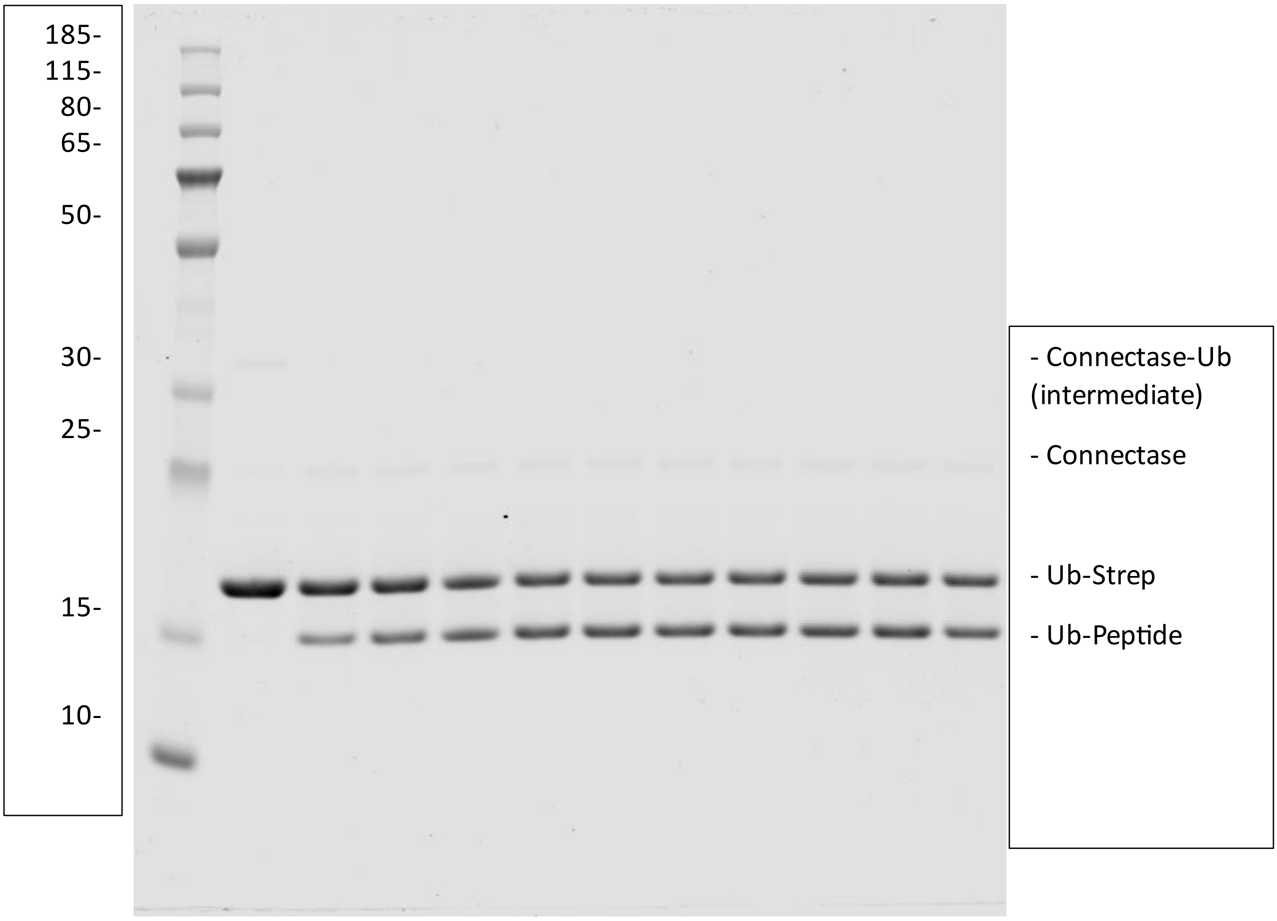

Lane	1	2	3	4	5	6	7	8	9	10	11
Marker	0	0.1	0.3	0.5	1	2	4	8	24	28	96
	Time [h]										

Gel 14 (Q)

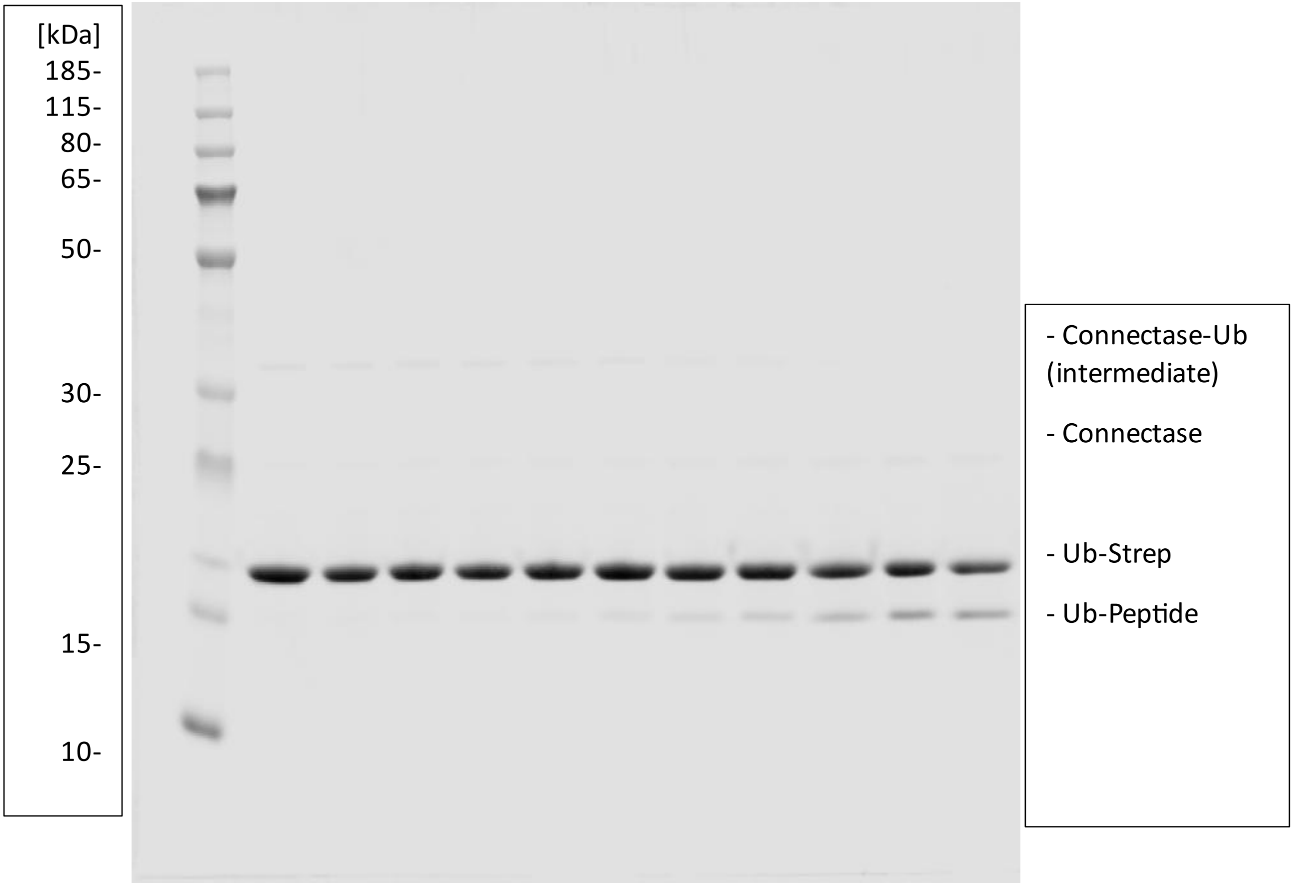

Lane	1	2	3	4	5	6	7	8	9	10	11
Marker	0	0.1	0.3	0.5	1	2	4	8	24	28	96
	Time [h]										

Gel 15 (R)

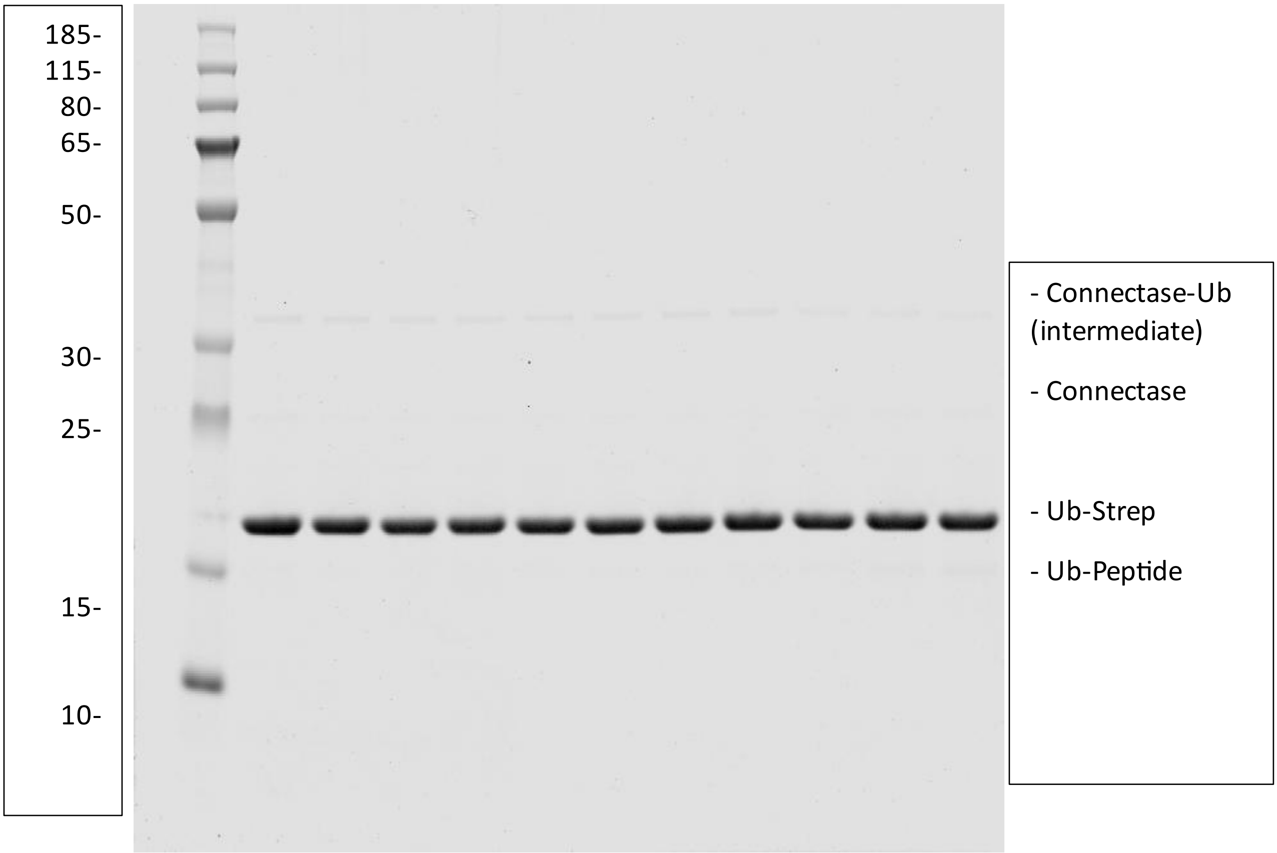

Lane	1	2	3	4	5	6	7	8	9	10	11
Marker	0	0.1	0.3	0.5	1	2	4	8	24	28	96
	Time [h]										

Gel 16 (S)

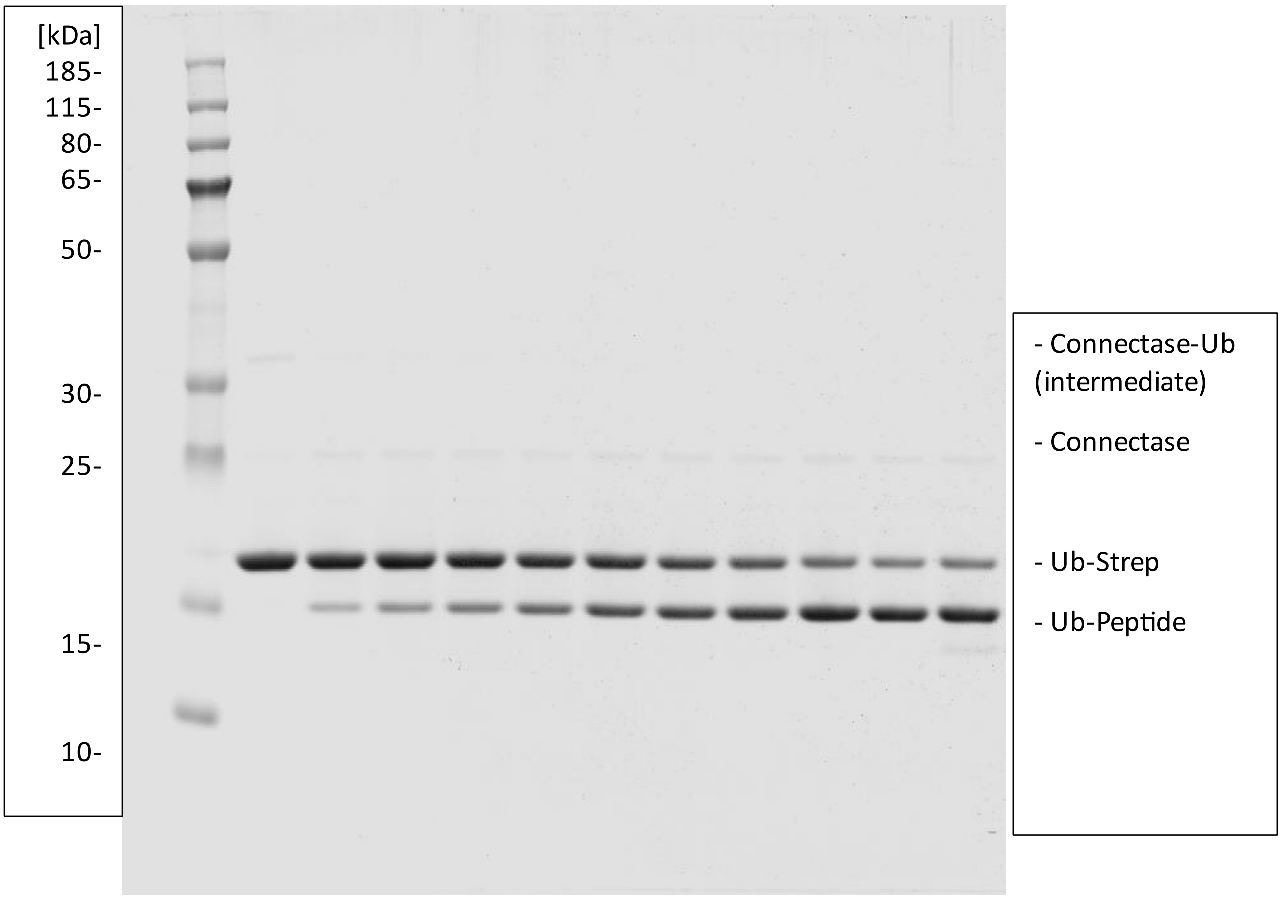

Lane	1	2	3	4	5	6	7	8	9	10	11
Marker	0	0.1	0.3	0.5	1	2	4	8	24	28	96
	Time [h]										

Gel 17 (T)

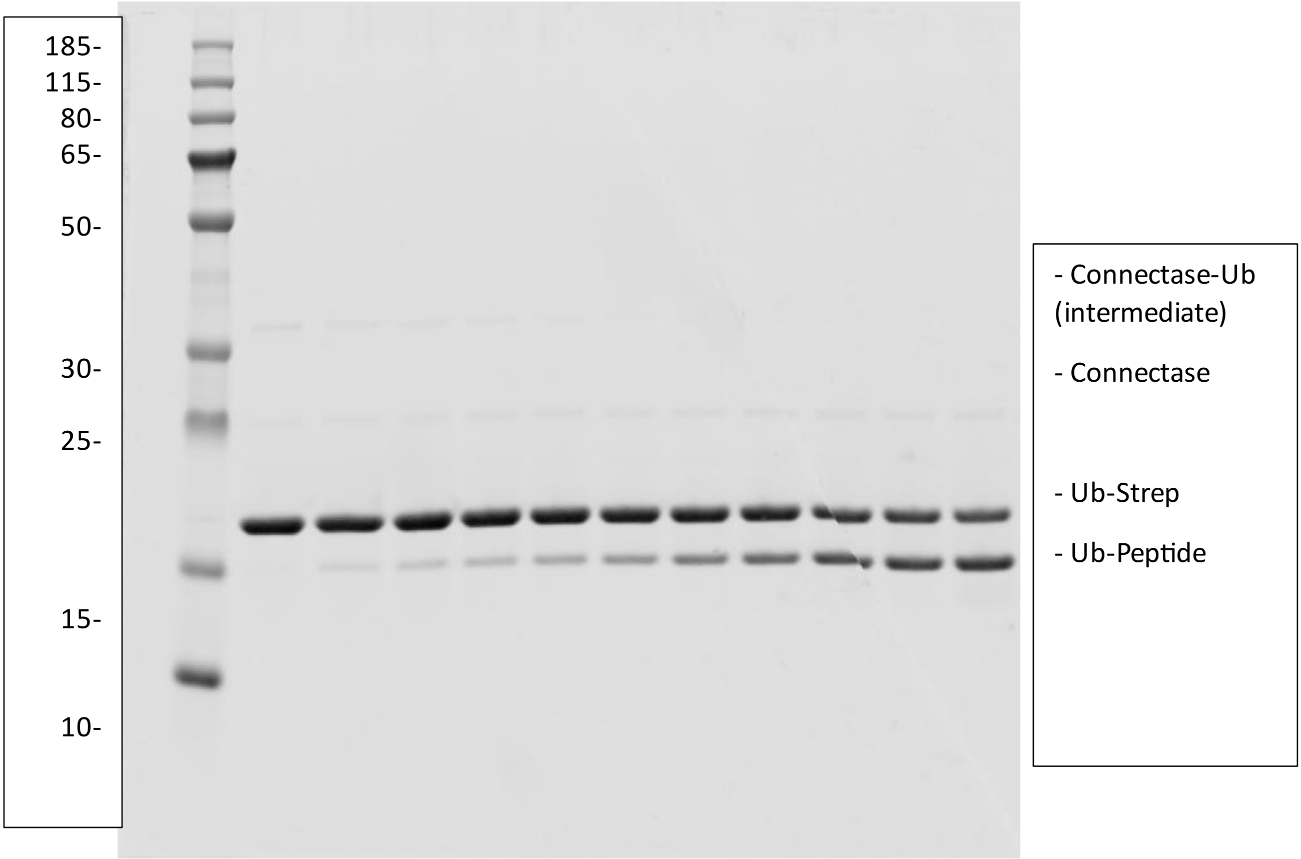

Lane	1	2	3	4	5	6	7	8	9	10	11
Marker	0	0.1	0.3	0.5	1	2	4	8	24	28	96
	Time [h]										

Gel 18 (V)

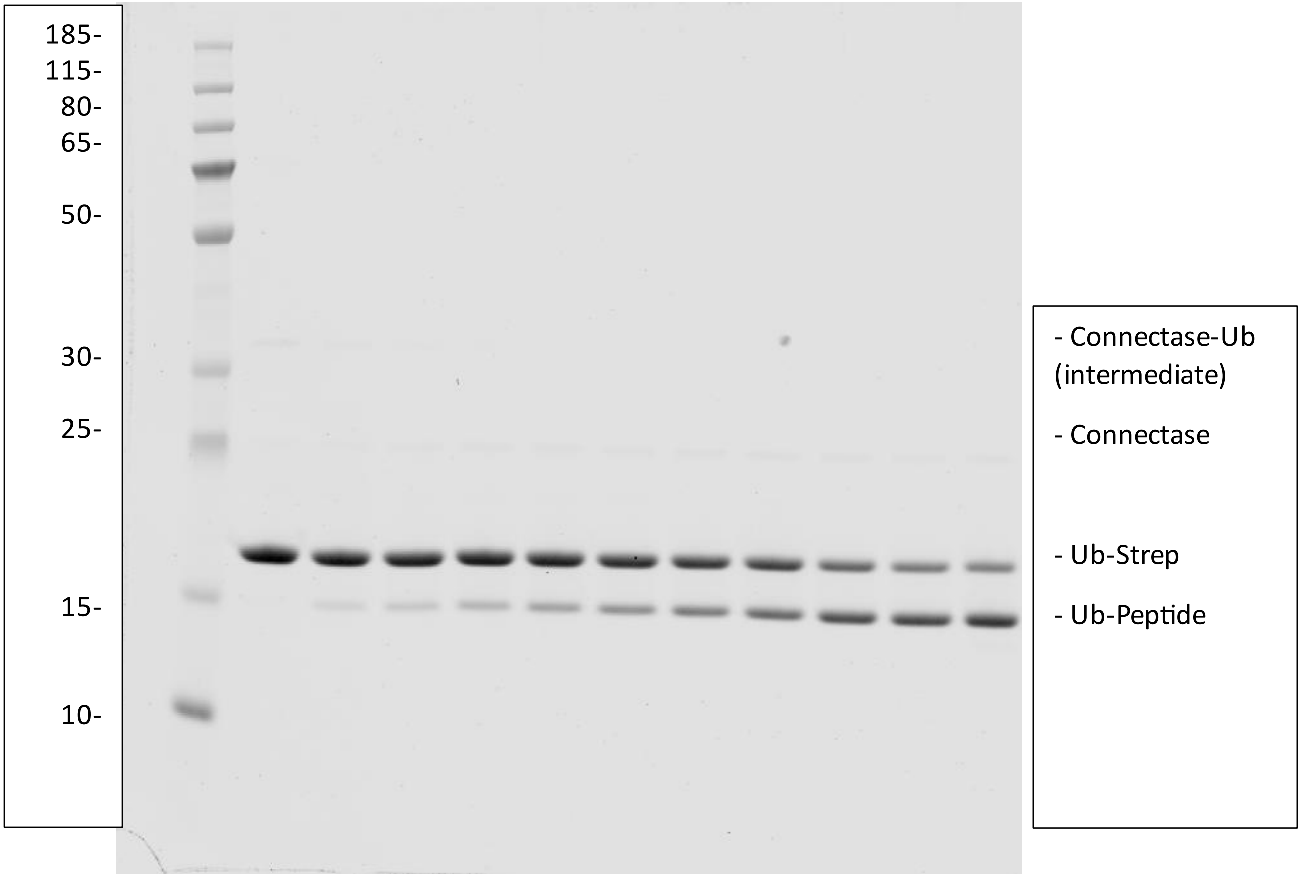

Lane	1	2	3	4	5	6	7	8	9	10	11
Marker	0	0.1	0.3	0.5	1	2	4	8	24	28	96
	Time [h]										

Gel 19 (W)

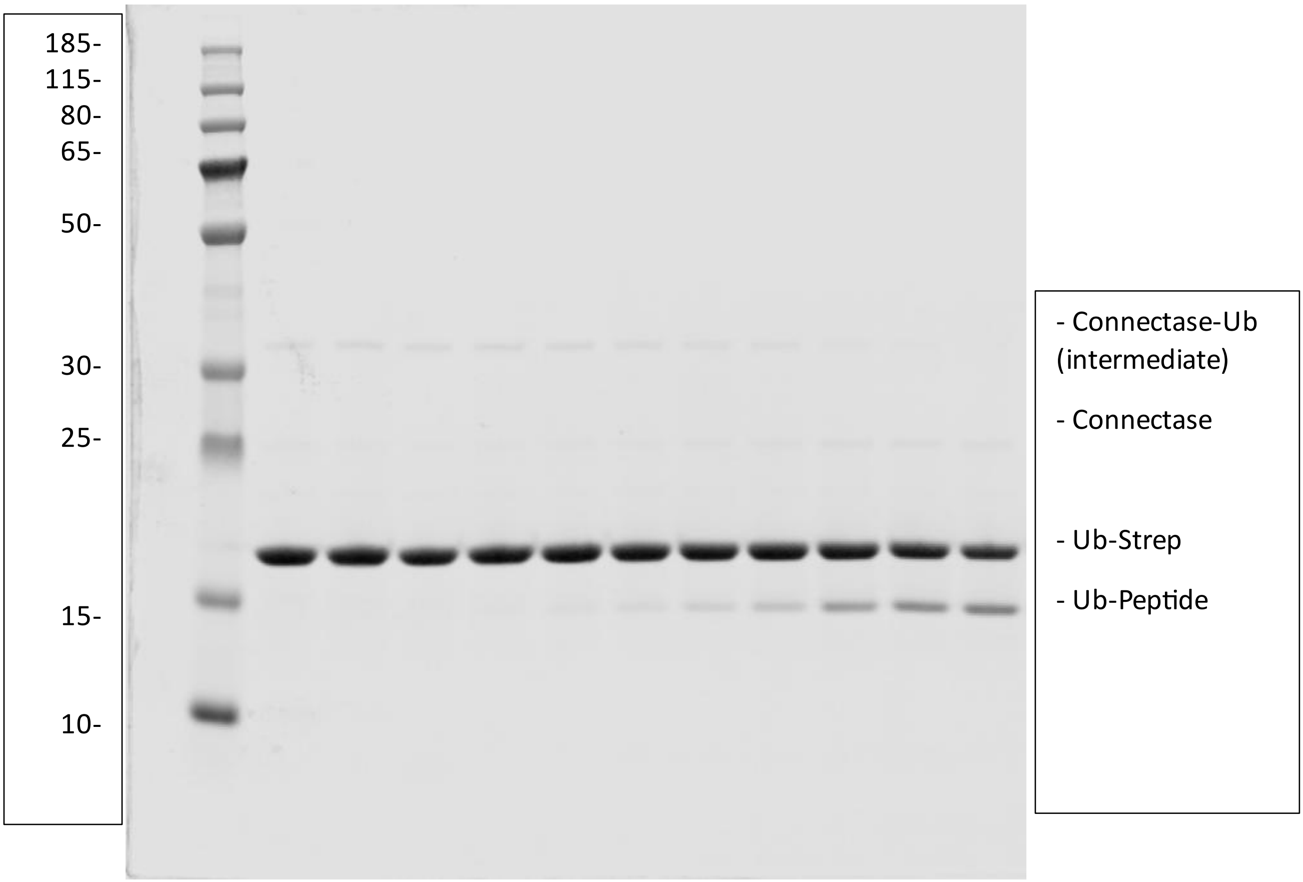

Lane	1	2	3	4	5	6	7	8	9	10	11
Marker	0	0.1	0.3	0.5	1	2	4	8	24	28	96
	Time [h]										

Gel 20 (Y)

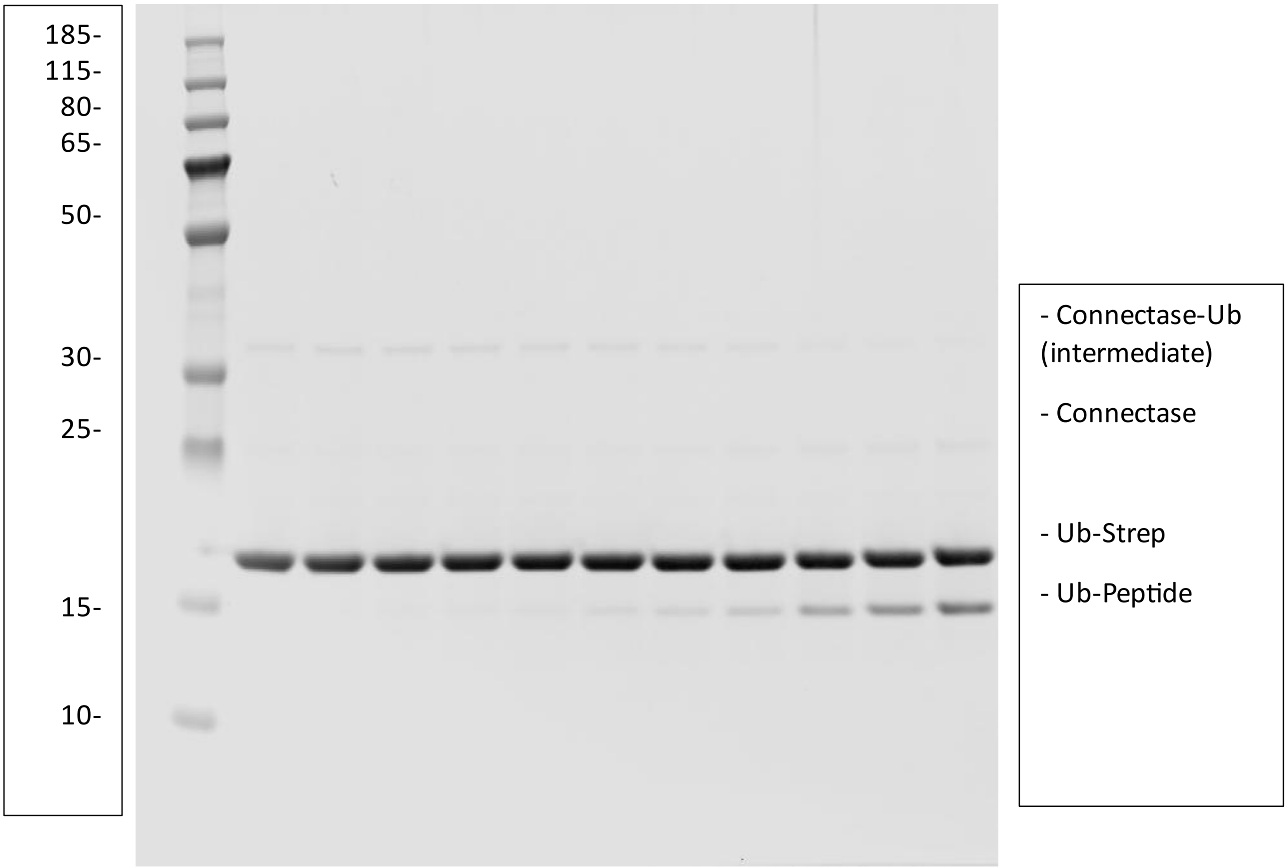

Lane	1	2	3	4	5	6	7	8	9	10	11
Marker	0	0.1	0.3	0.5	1	2	4	8	24	28	96
	Time [h]										
