# Supplementary figures and images for: Enzymatic protein fusions with 100% product yield
Source: eLife. 2025 Apr 1;13:RP102765. doi: 10.7554/eLife.102765 (PMC11961121; doi:10.7554/eLife.102765)

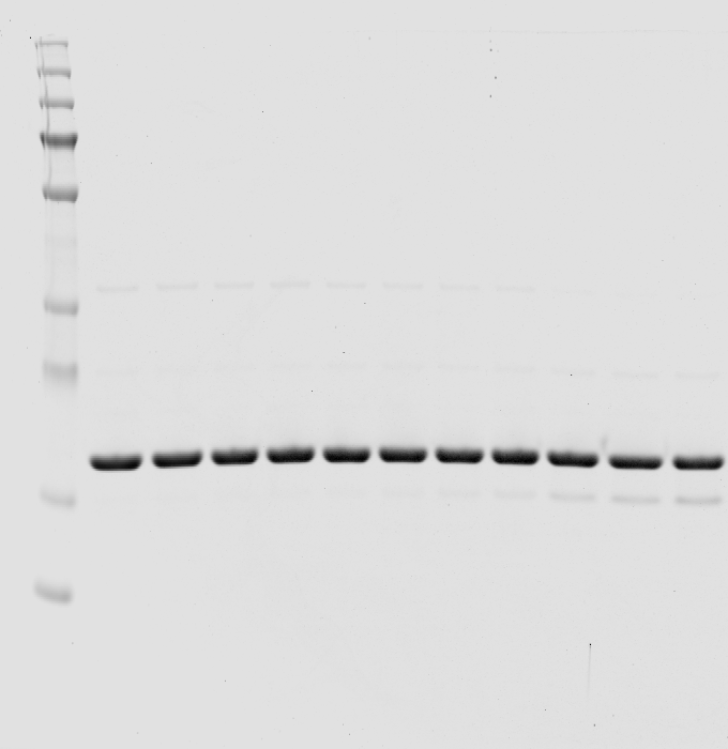

Supplement: Figure 2—source data 2. [file elife-102765-fig2-data2.zip › Figure 2 - Source Data 12 - N.tif]

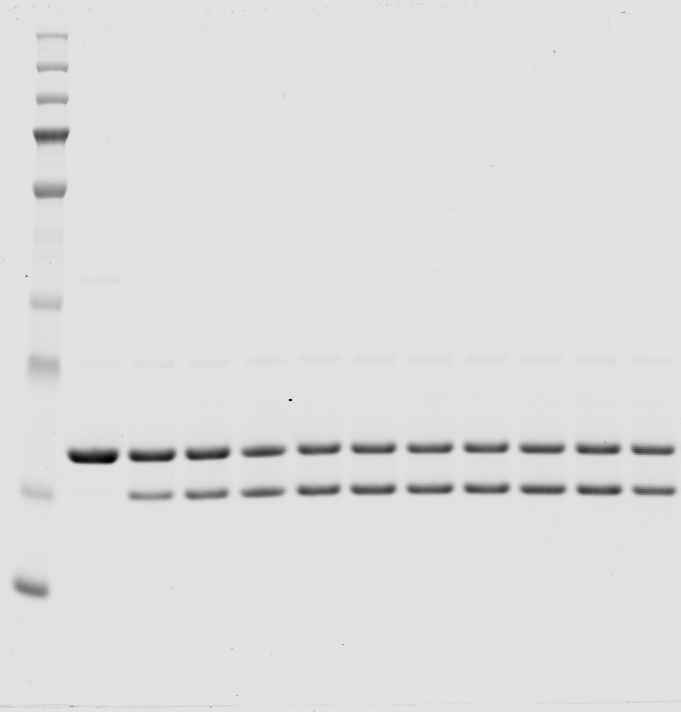

Supplement: Figure 2—source data 2. [file elife-102765-fig2-data2.zip › Figure 2 - Source Data 13 - P.tif]

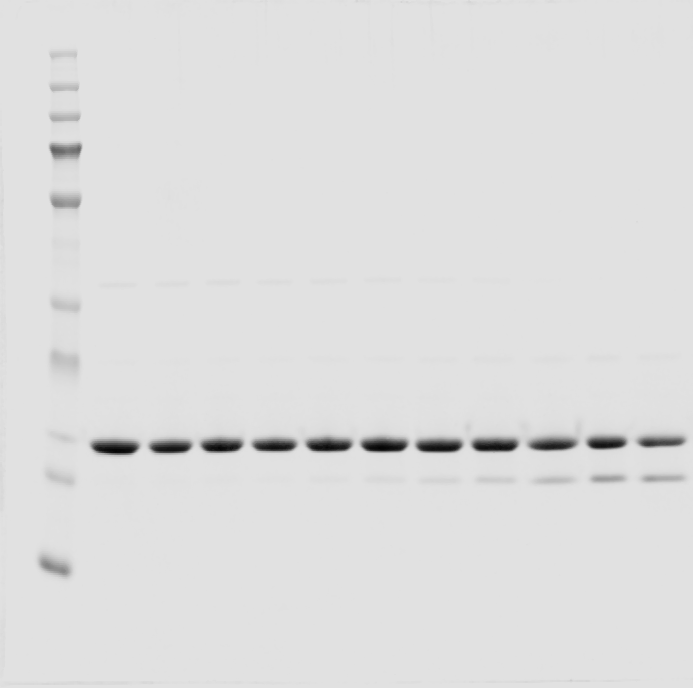

Supplement: Figure 2—source data 2. [file elife-102765-fig2-data2.zip › Figure 2 - Source Data 14 - Q.tif]

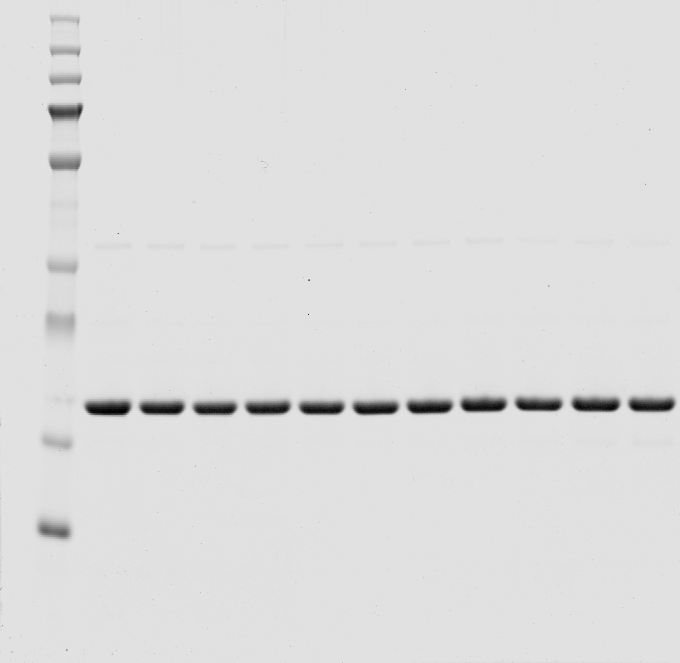

Supplement: Figure 2—source data 2. [file elife-102765-fig2-data2.zip › Figure 2 - Source Data 15 - R.tif]

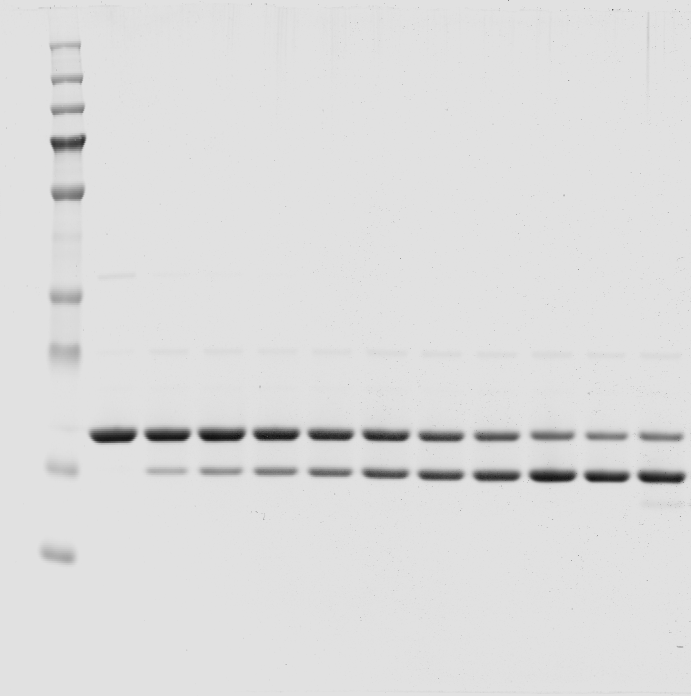

Supplement: Figure 2—source data 2. [file elife-102765-fig2-data2.zip › Figure 2 - Source Data 16 - S.tif]

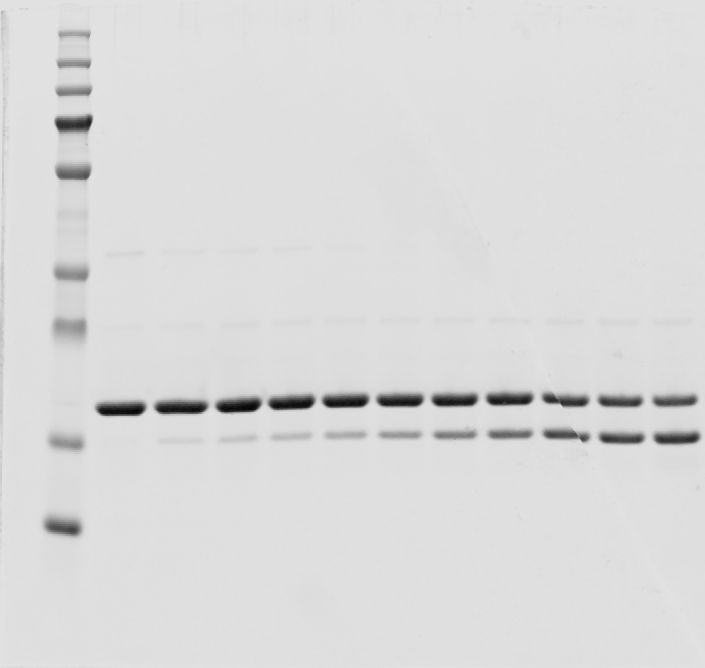

Supplement: Figure 2—source data 2. [file elife-102765-fig2-data2.zip › Figure 2 - Source Data 17 - T.tif]

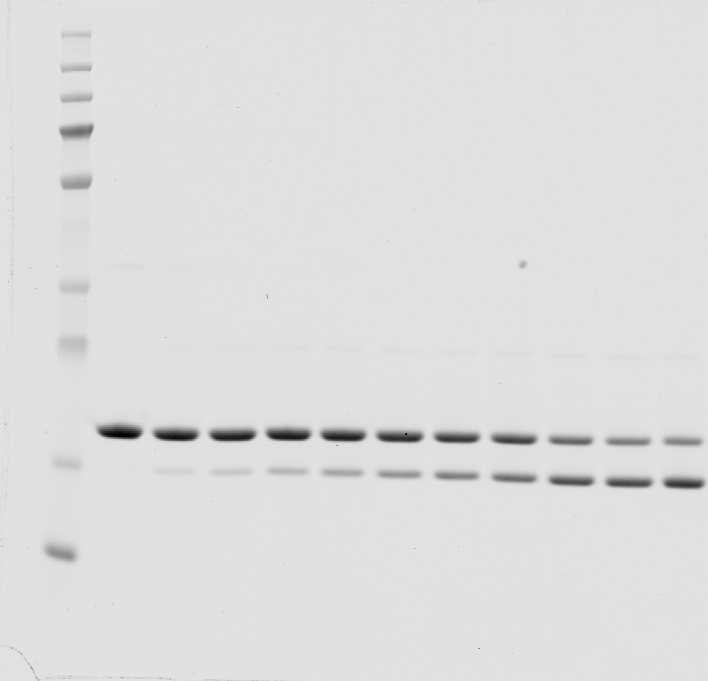

Supplement: Figure 2—source data 2. [file elife-102765-fig2-data2.zip › Figure 2 - Source Data 18 - V.tif]

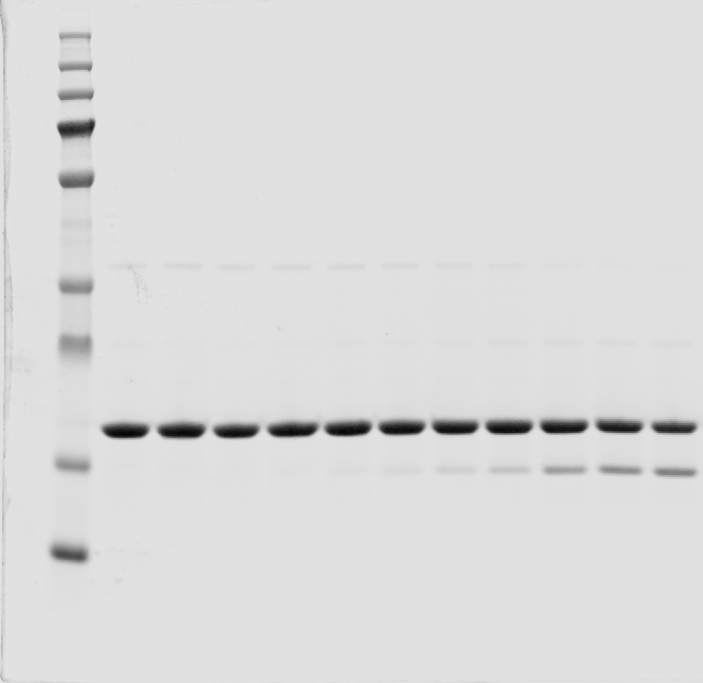

Supplement: Figure 2—source data 2. [file elife-102765-fig2-data2.zip › Figure 2 - Source Data 19 - W.tif]

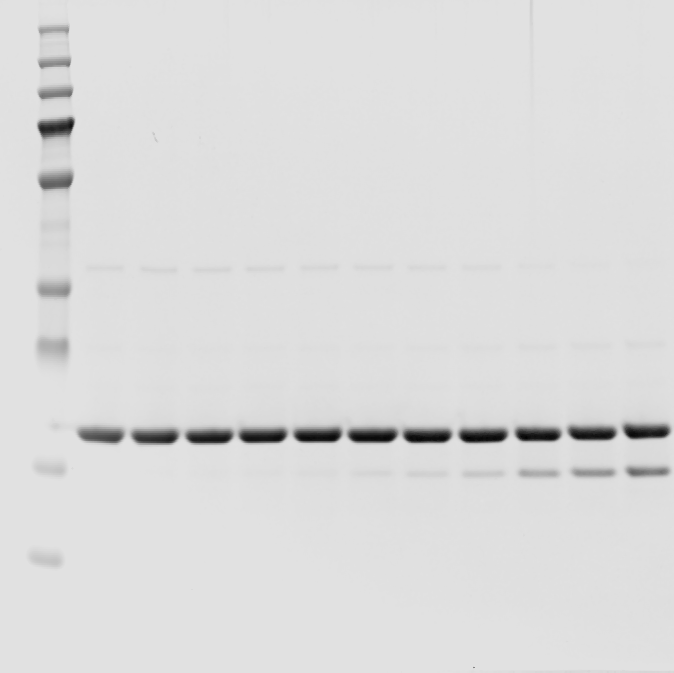

Supplement: Figure 2—source data 2. [file elife-102765-fig2-data2.zip › Figure 2 - Source Data 20 - Y.tif]

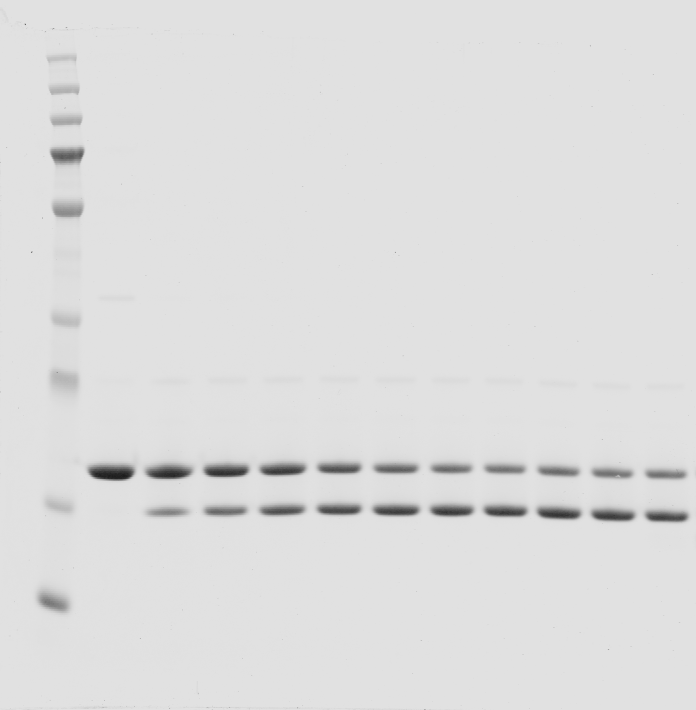

Supplement: Figure 2—source data 2. [file elife-102765-fig2-data2.zip › Figure 2 - Source Data 1 - A.tif]

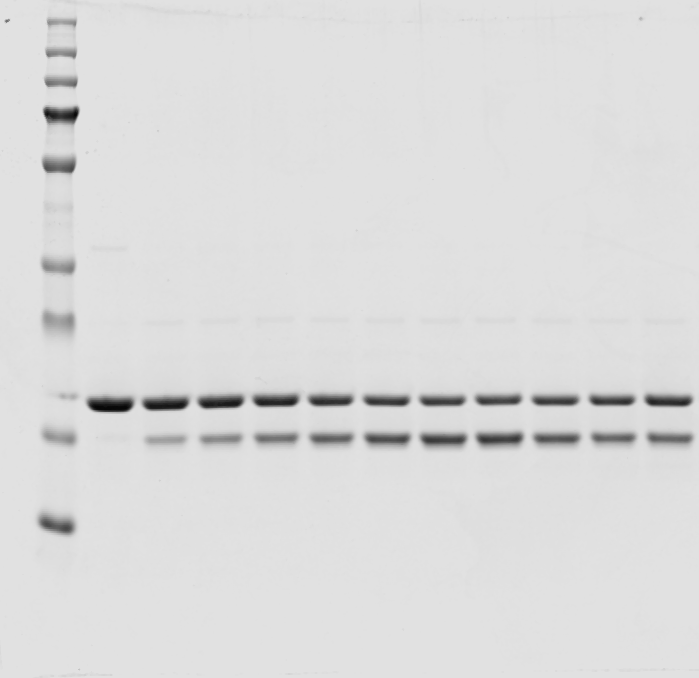

Supplement: Figure 2—source data 2. [file elife-102765-fig2-data2.zip › Figure 2 - Source Data 2 - C.tif]

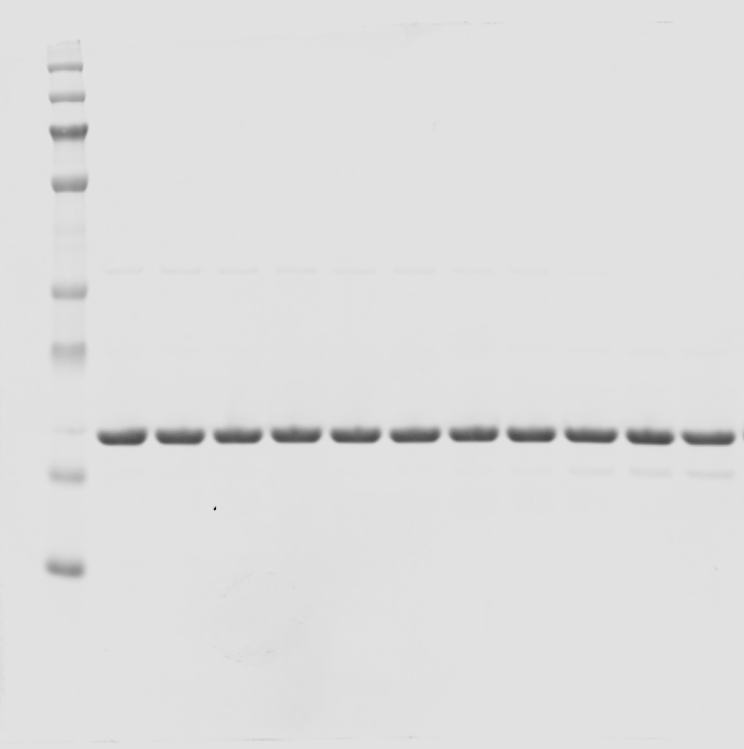

Supplement: Figure 2—source data 2. [file elife-102765-fig2-data2.zip › Figure 2 - Source Data 3 - D.tif]

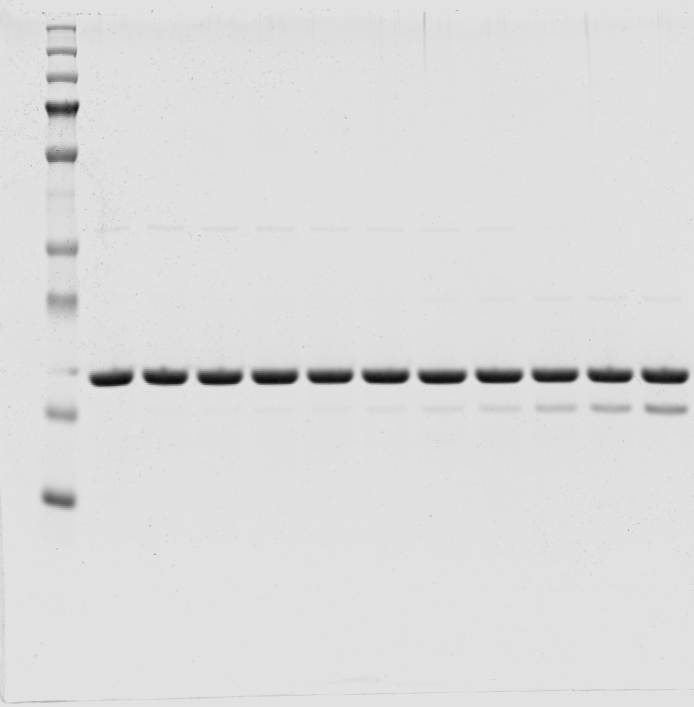

Supplement: Figure 2—source data 2. [file elife-102765-fig2-data2.zip › Figure 2 - Source Data 4 - E.tif]

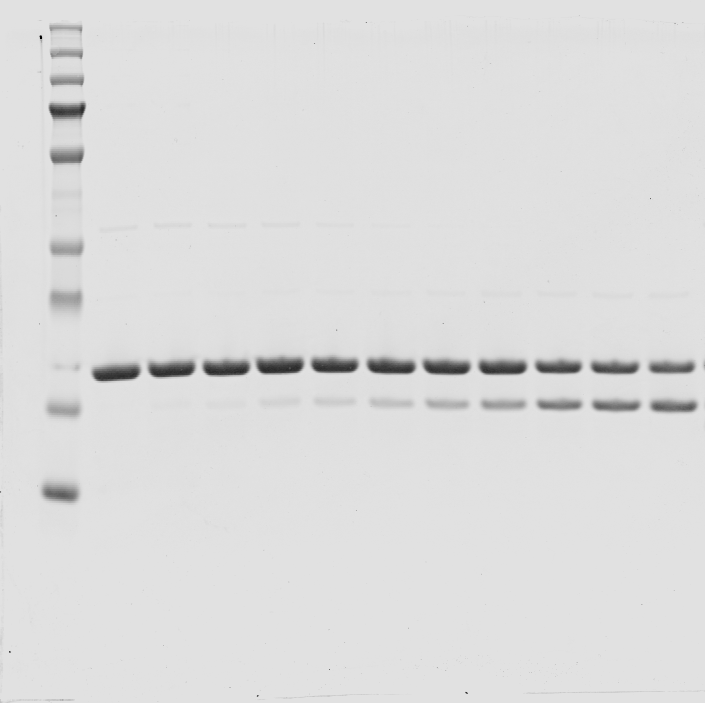

Supplement: Figure 2—source data 2. [file elife-102765-fig2-data2.zip › Figure 2 - Source Data 5 - F.tif]

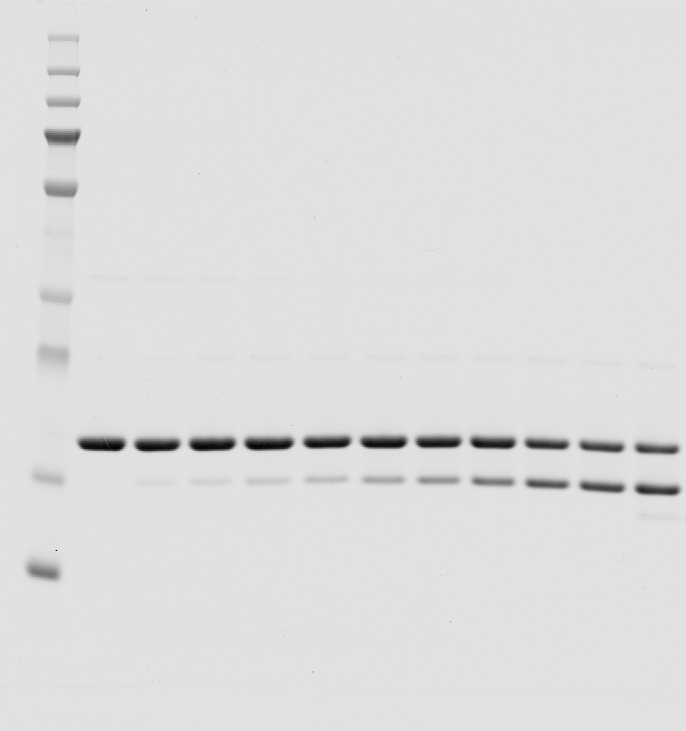

Supplement: Figure 2—source data 2. [file elife-102765-fig2-data2.zip › Figure 2 - Source Data 6 - G.tif]

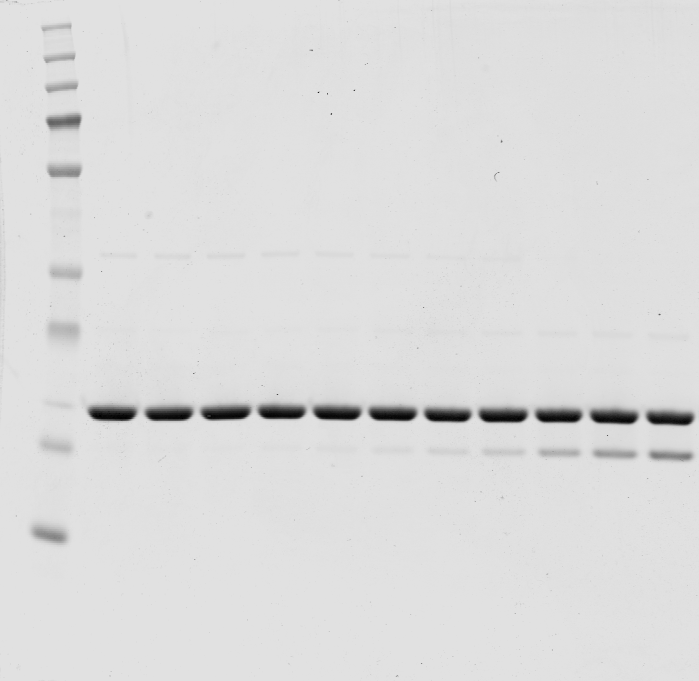

Supplement: Figure 2—source data 2. [file elife-102765-fig2-data2.zip › Figure 2 - Source Data 7 - H.tif]

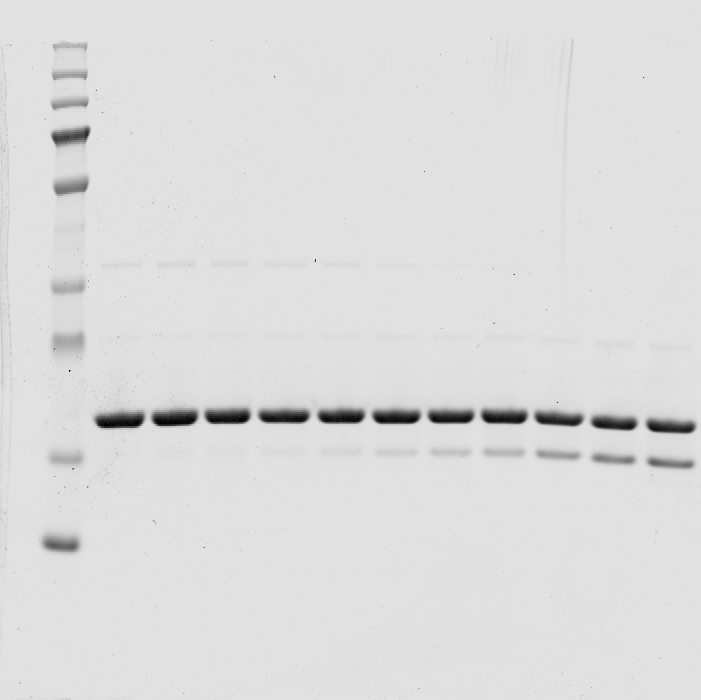

Supplement: Figure 2—source data 2. [file elife-102765-fig2-data2.zip › Figure 2 - Source Data 8 - I.tif]

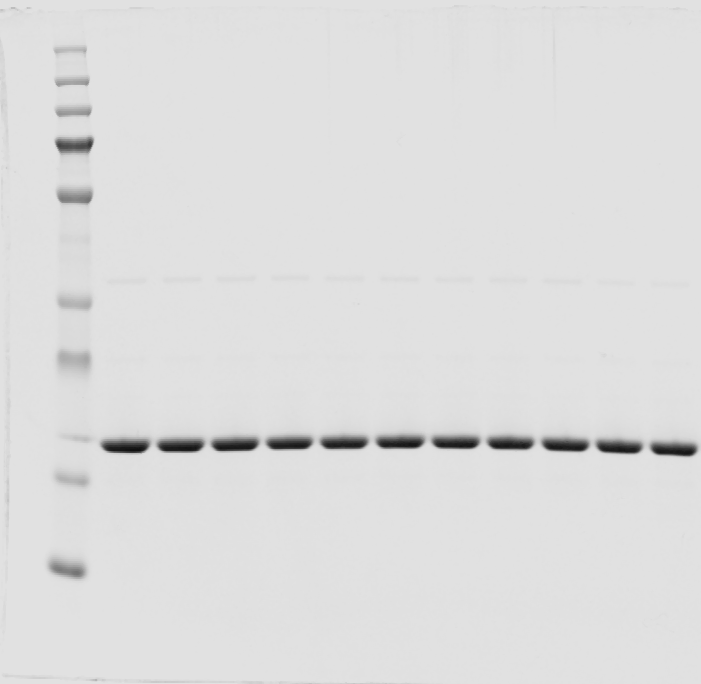

Supplement: Figure 2—source data 2. [file elife-102765-fig2-data2.zip › Figure 2 - Source Data 9 - K.tif]

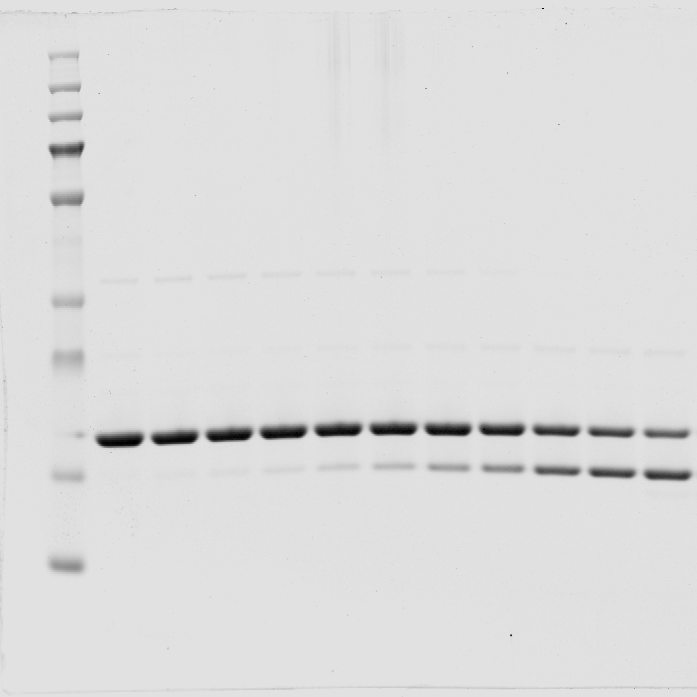

Supplement: Figure 2—source data 2. [file elife-102765-fig2-data2.zip › Figure 2 - Source Data 10 - L.tif]

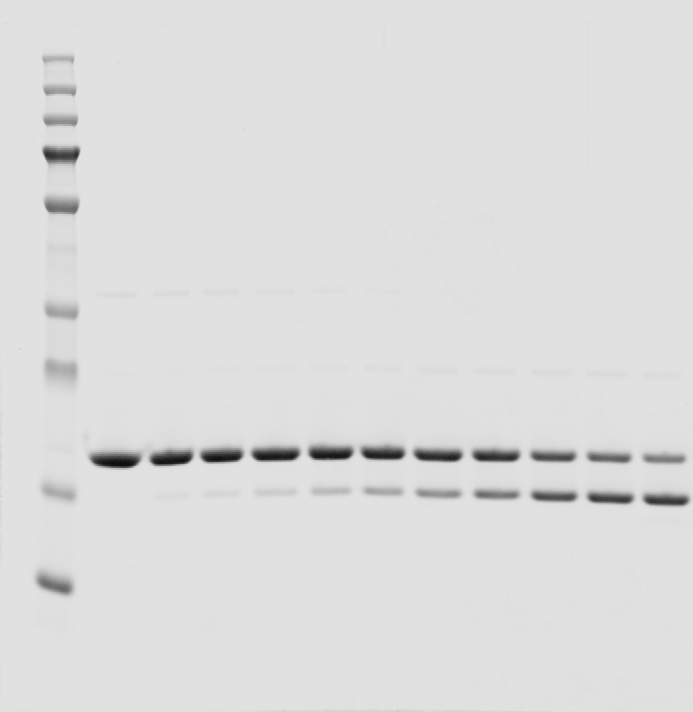

Supplement: Figure 2—source data 2. [file elife-102765-fig2-data2.zip › Figure 2 - Source Data 11 - M.tif]

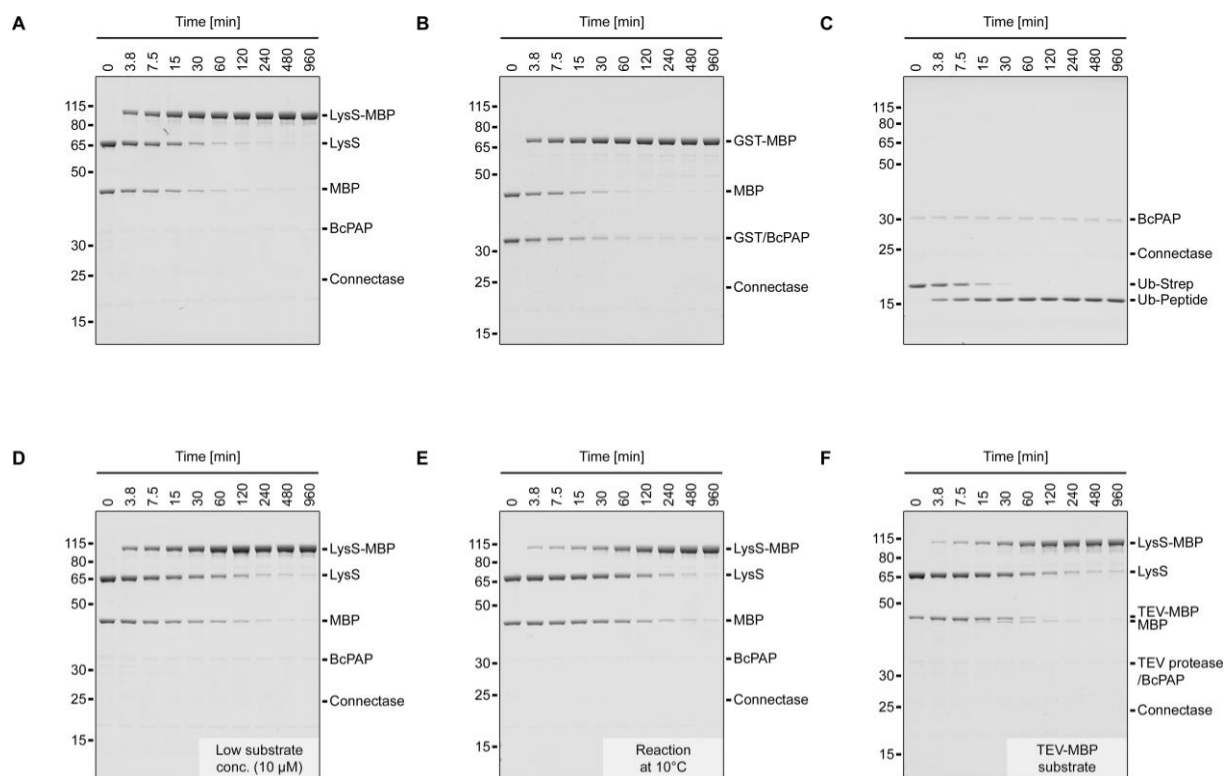

Supplement: Figure 3—source data 1. [file elife-102765-fig3-data1.pdf]

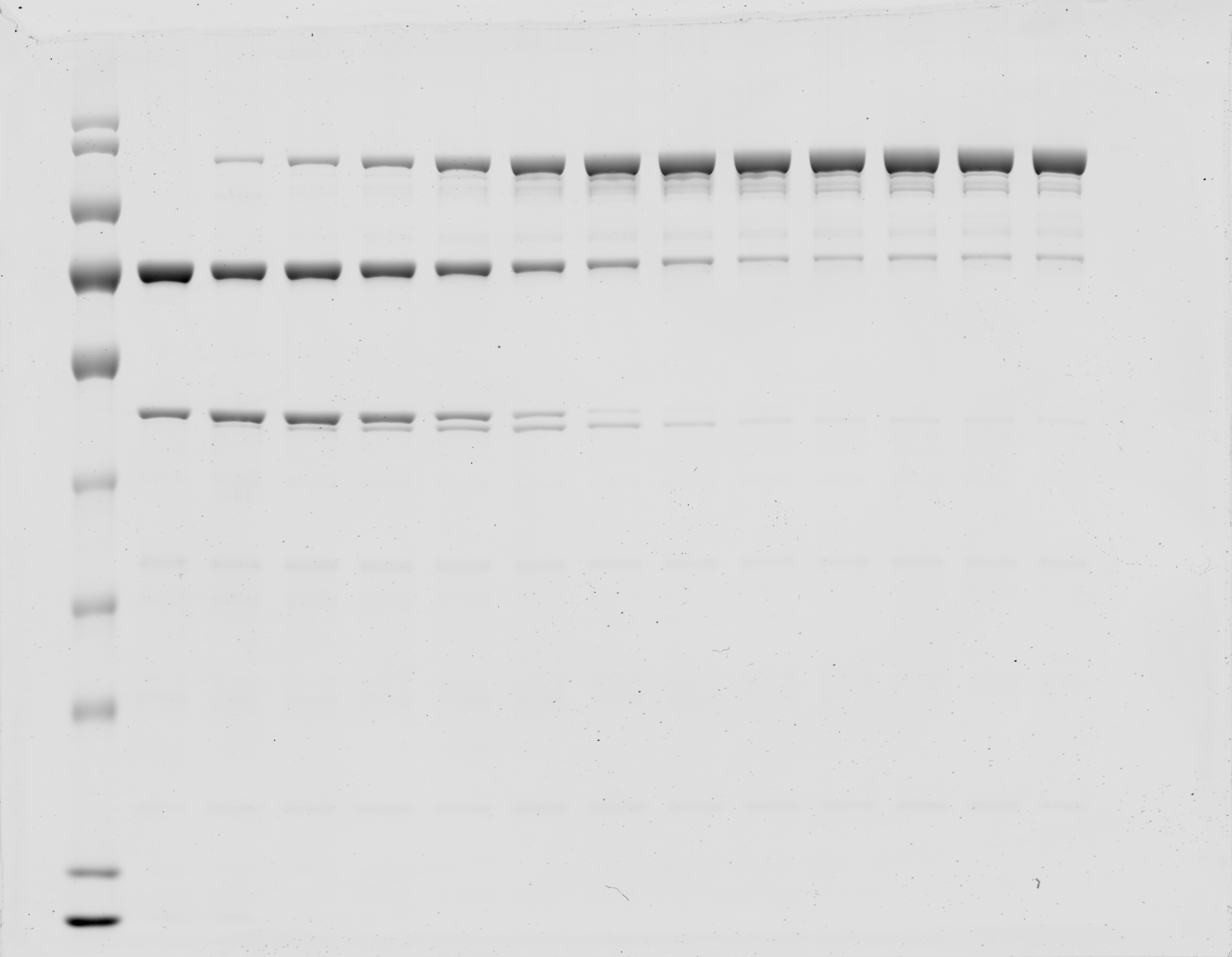

Supplement: Figure 3—source data 2. [file elife-102765-fig3-data2.zip › Figure 3F - Source Data.tif]

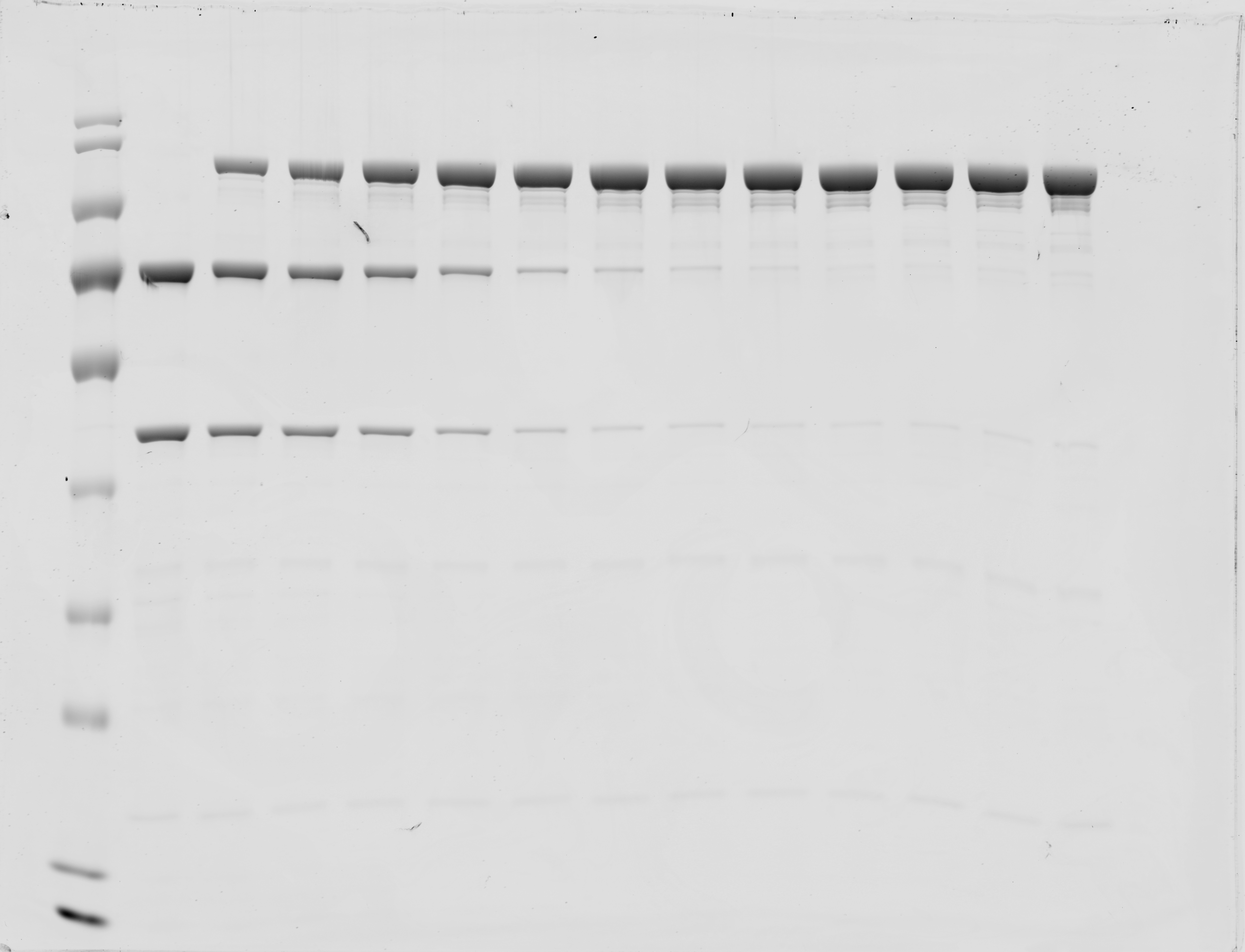

Supplement: Figure 3—source data 2. [file elife-102765-fig3-data2.zip › Figure 3A - Source Data.tif]

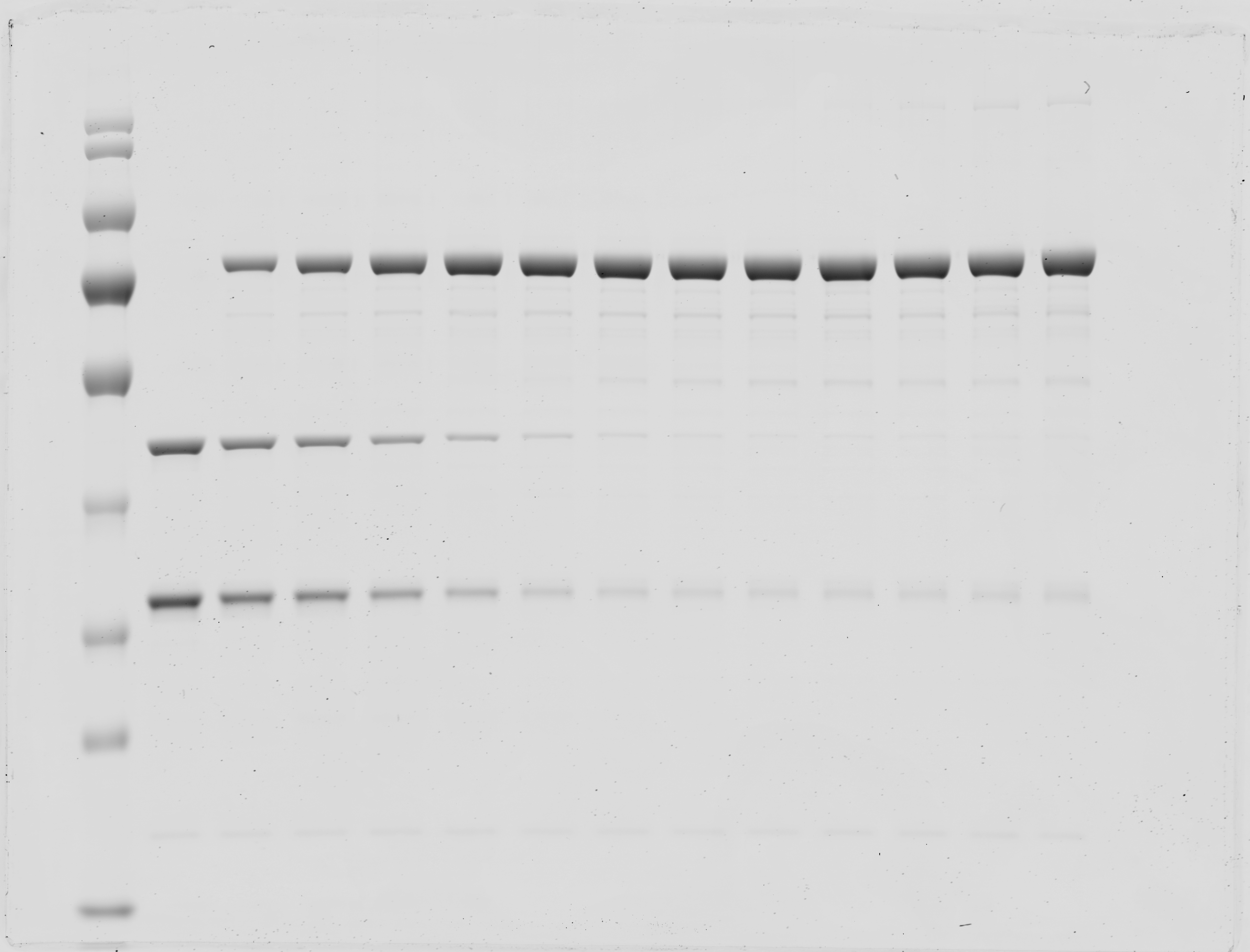

Supplement: Figure 3—source data 2. [file elife-102765-fig3-data2.zip › Figure 3B - Source Data.tif]

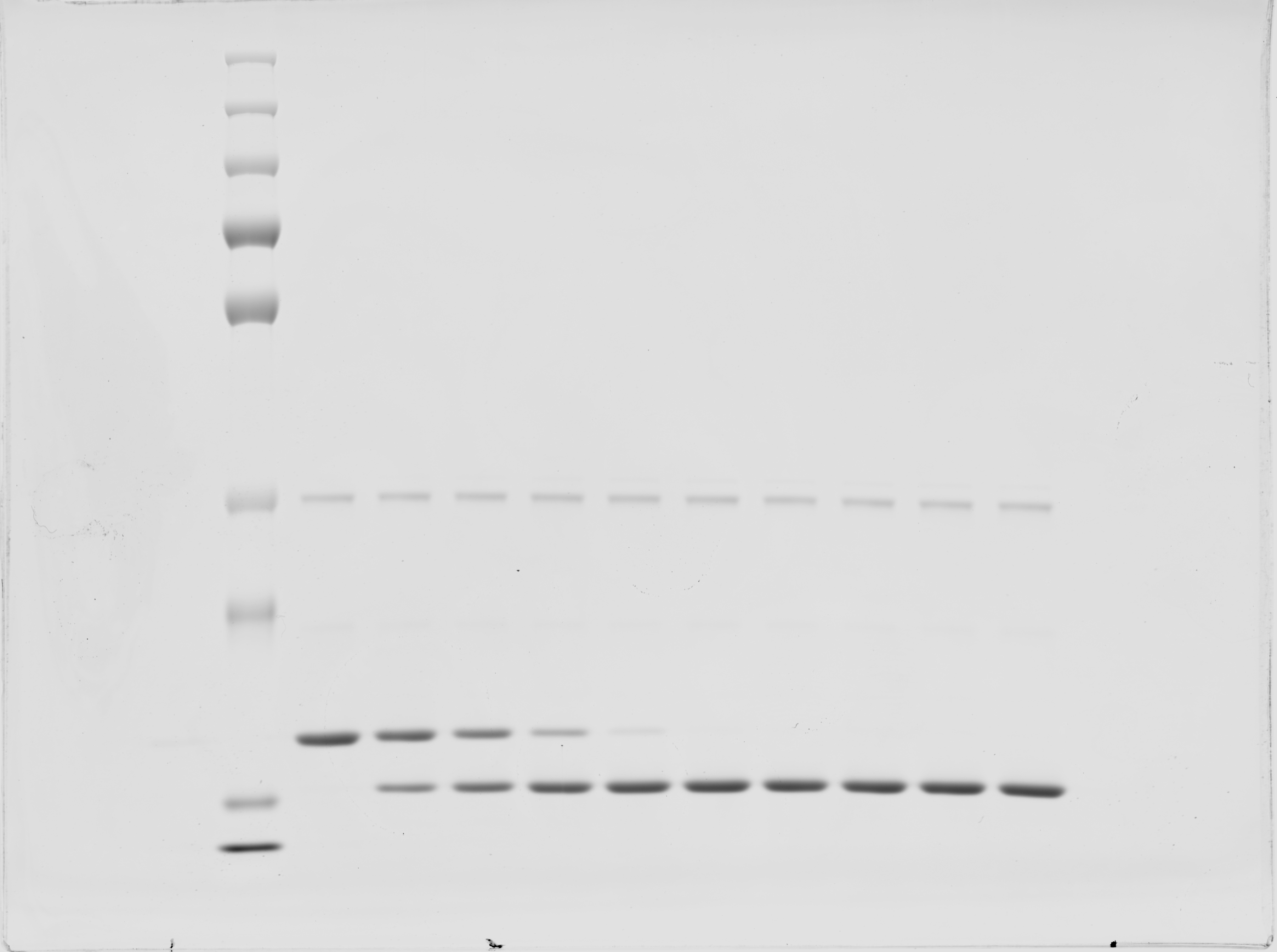

Supplement: Figure 3—source data 2. [file elife-102765-fig3-data2.zip › Figure 3C - Source Data.tif]

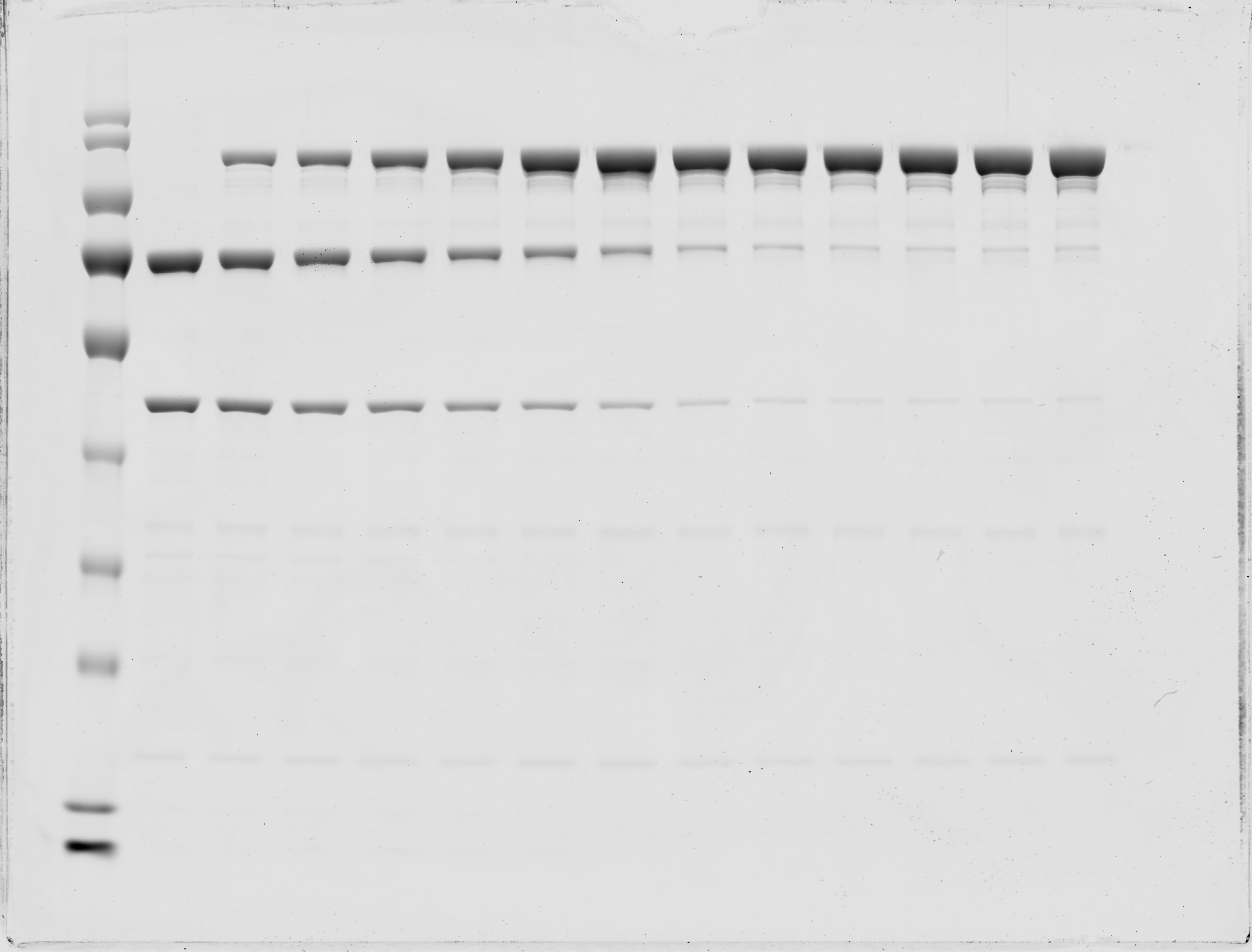

Supplement: Figure 3—source data 2. [file elife-102765-fig3-data2.zip › Figure 3D - Source Data.tif]

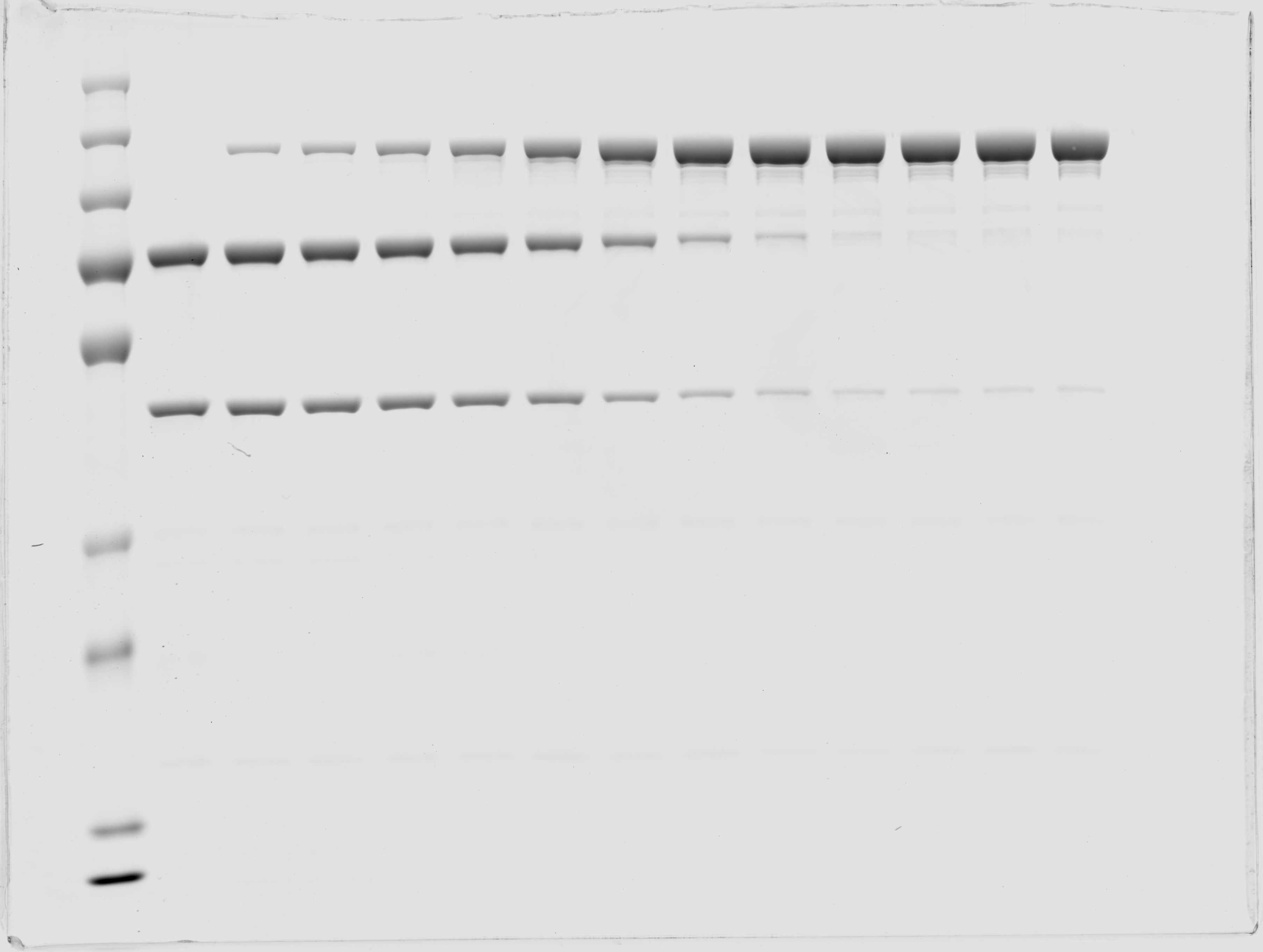

Supplement: Figure 3—source data 2. [file elife-102765-fig3-data2.zip › Figure 3E - Source Data.tif]

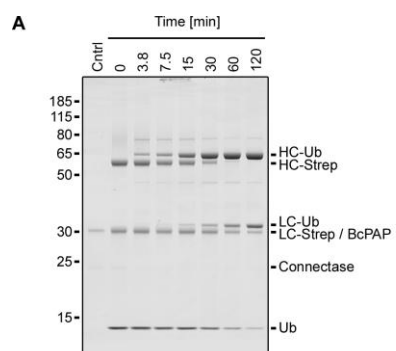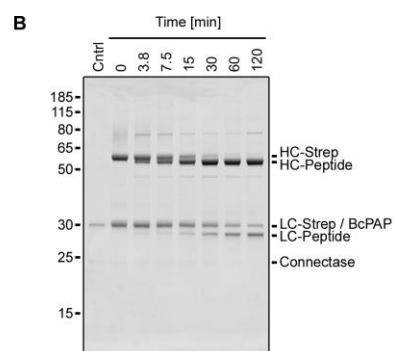

Supplement: Figure 4—source data 1. [file elife-102765-fig4-data1.pdf]

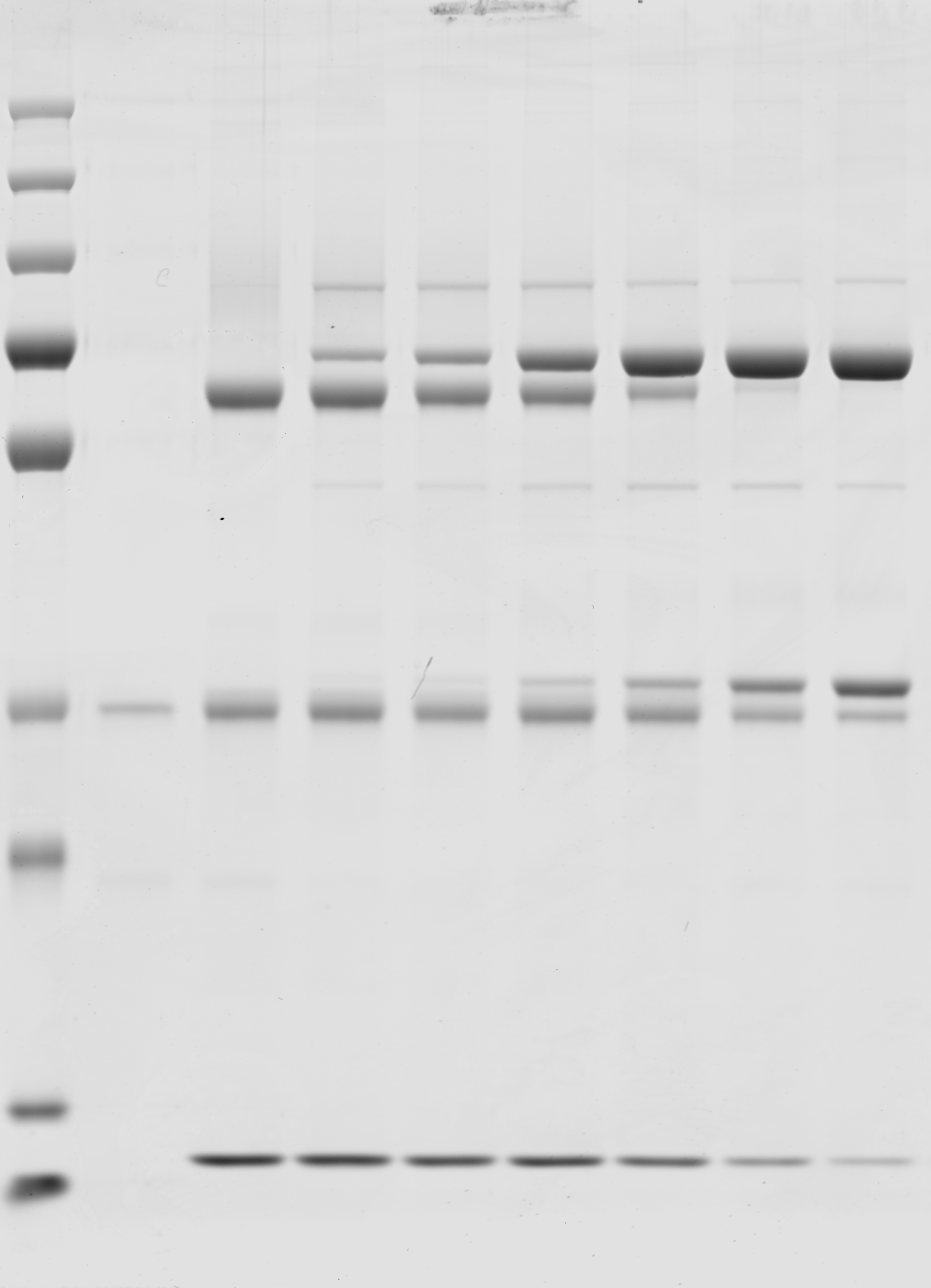

Supplement: Figure 4—source data 2. [file elife-102765-fig4-data2.zip › Figure 4A - Source Data.tif]

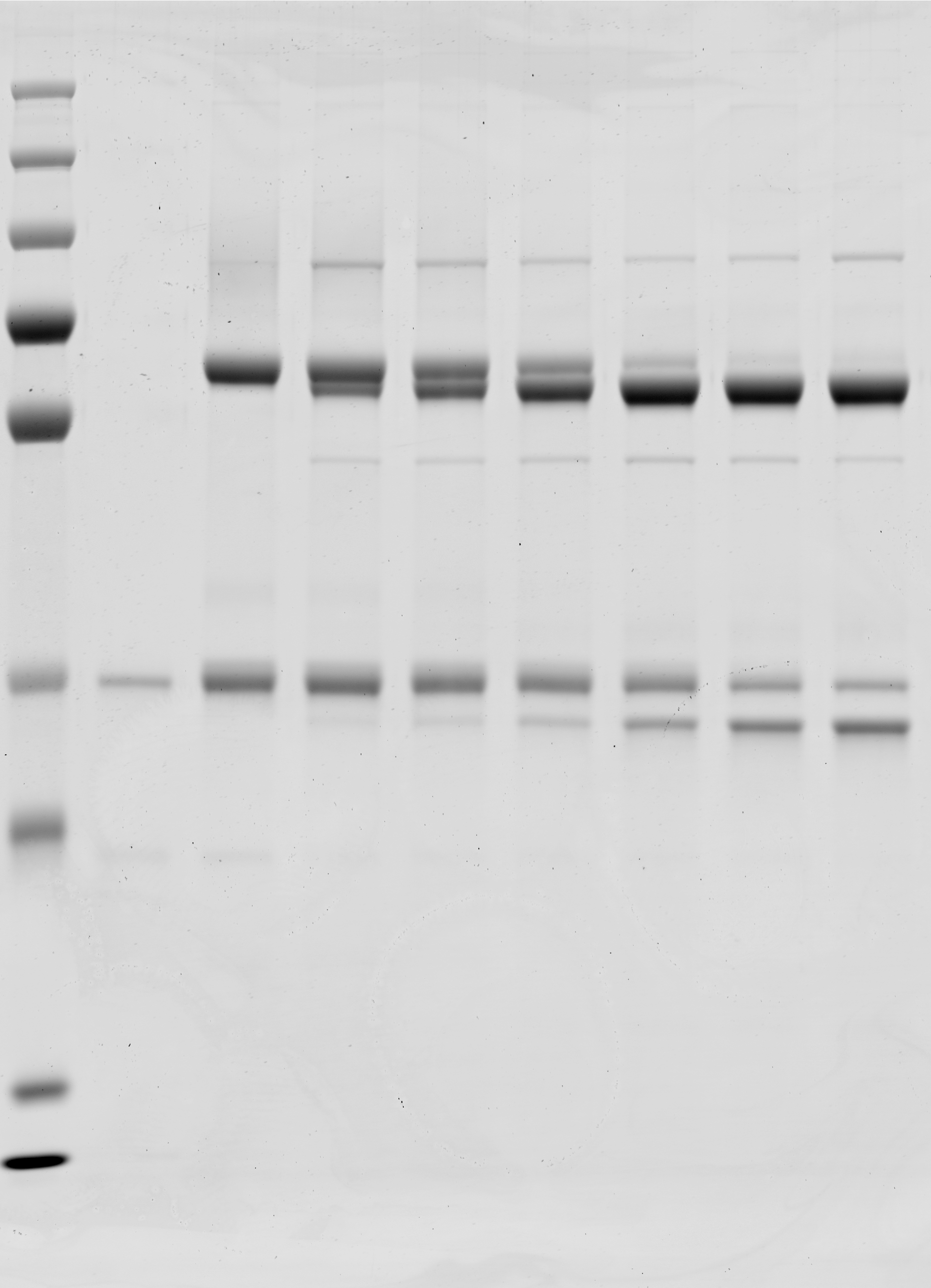

Supplement: Figure 4—source data 2. [file elife-102765-fig4-data2.zip › Figure 4B - Source Data.tif]

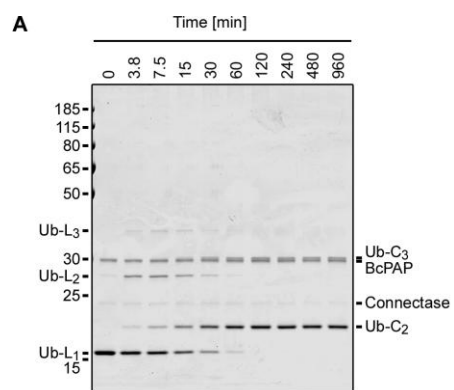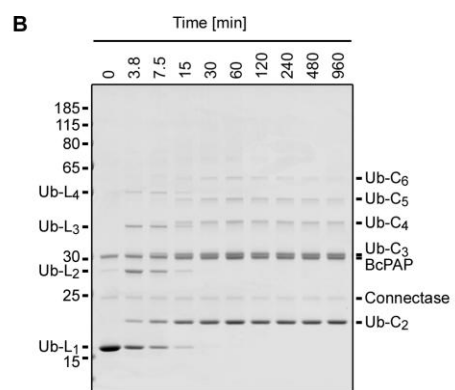

Supplement: Figure 5—source data 1. [file elife-102765-fig5-data1.pdf]

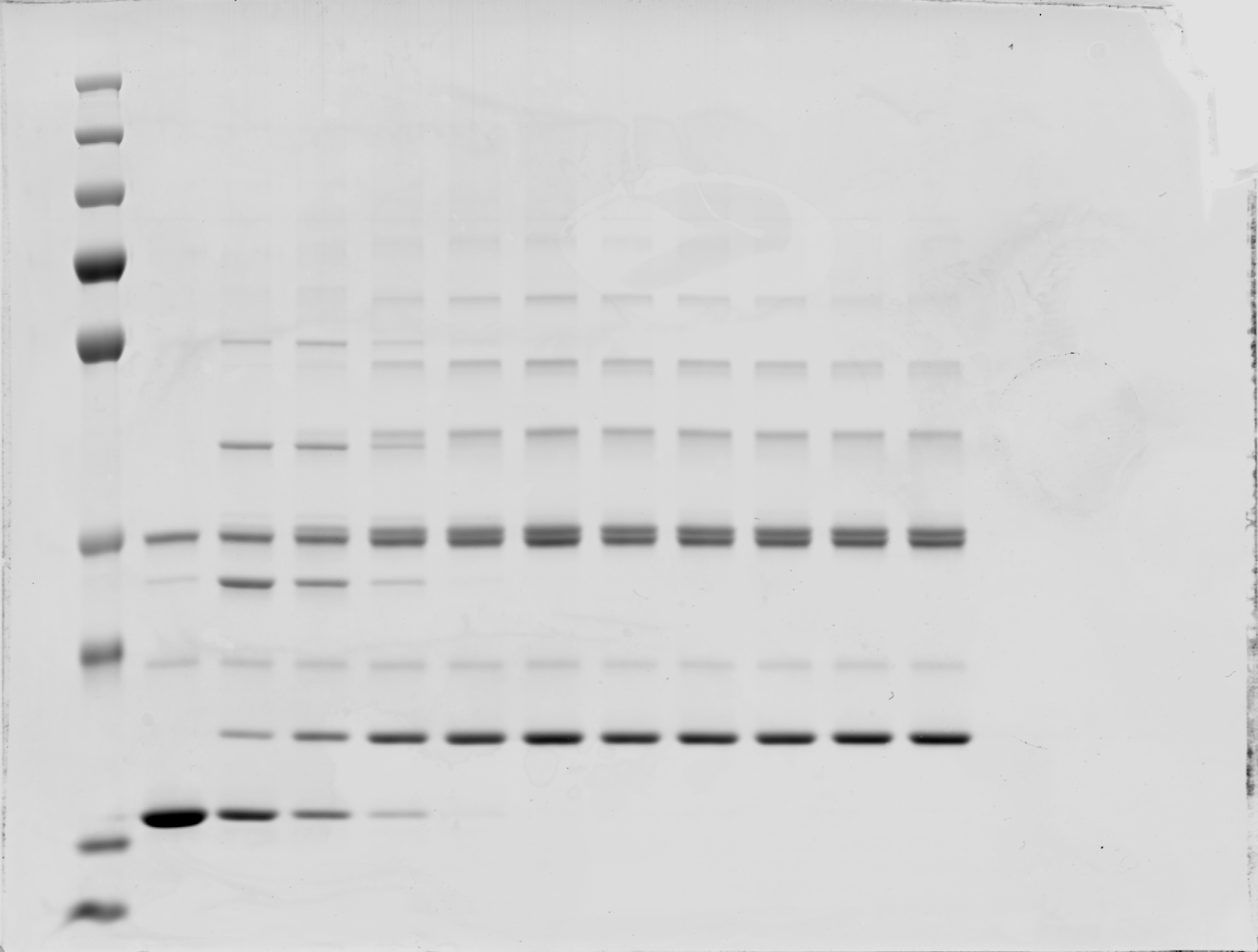

Supplement: Figure 5—source data 2. [file elife-102765-fig5-data2.zip › Figure 5B - Source Data.tif]

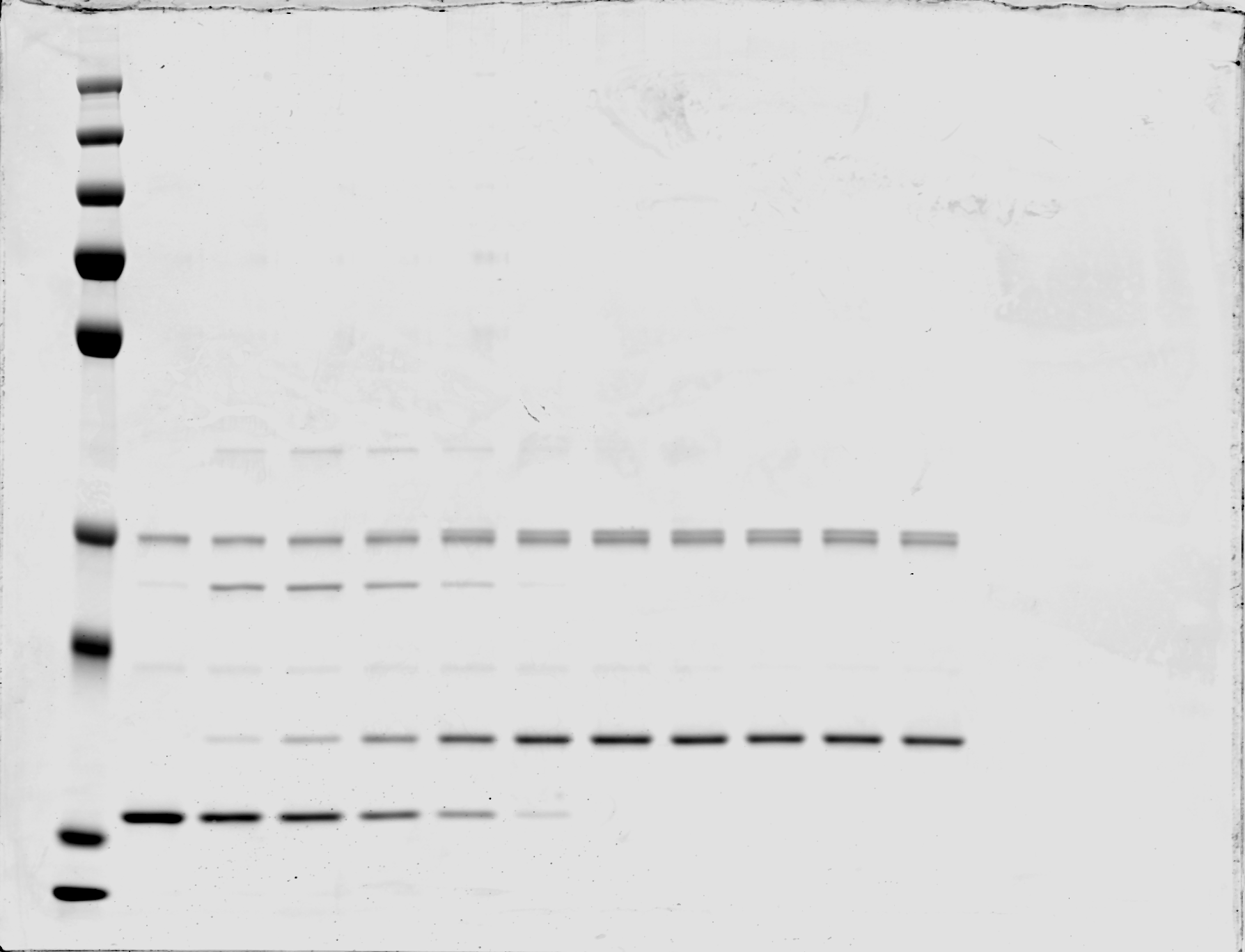

Supplement: Figure 5—source data 2. [file elife-102765-fig5-data2.zip › Figure 5A - Source Data.tif]

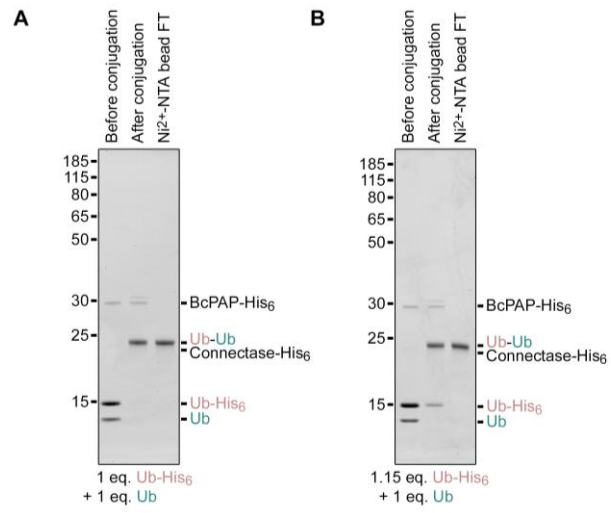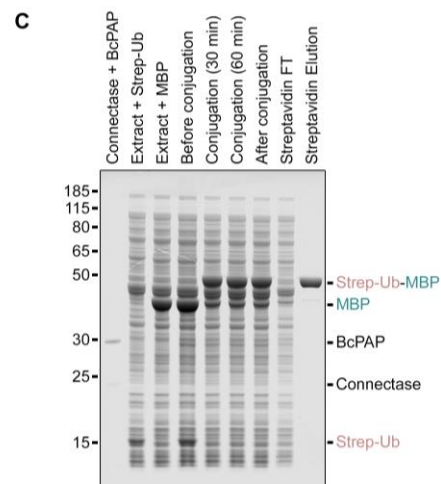

Supplement: Figure 6—source data 1. [file elife-102765-fig6-data1.pdf]

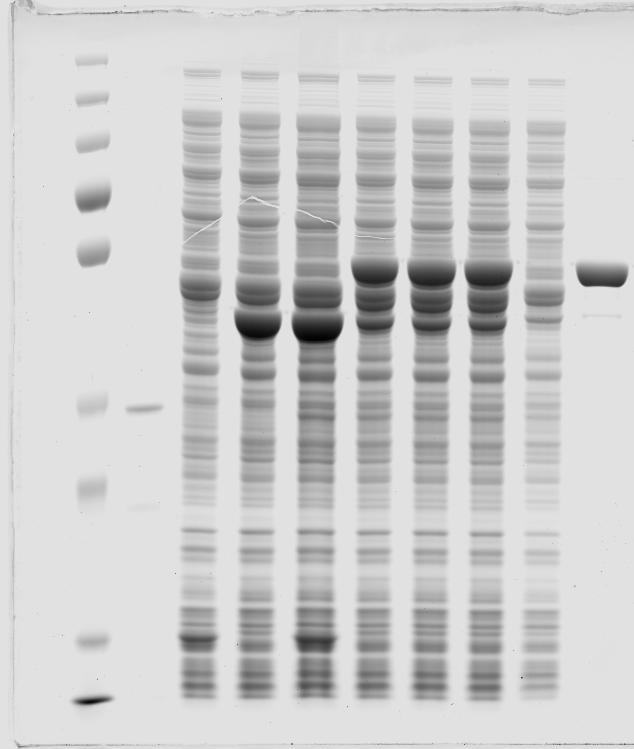

Supplement: Figure 6—source data 2. [file elife-102765-fig6-data2.zip › Figure 6C - Source Data.TIF.tif]

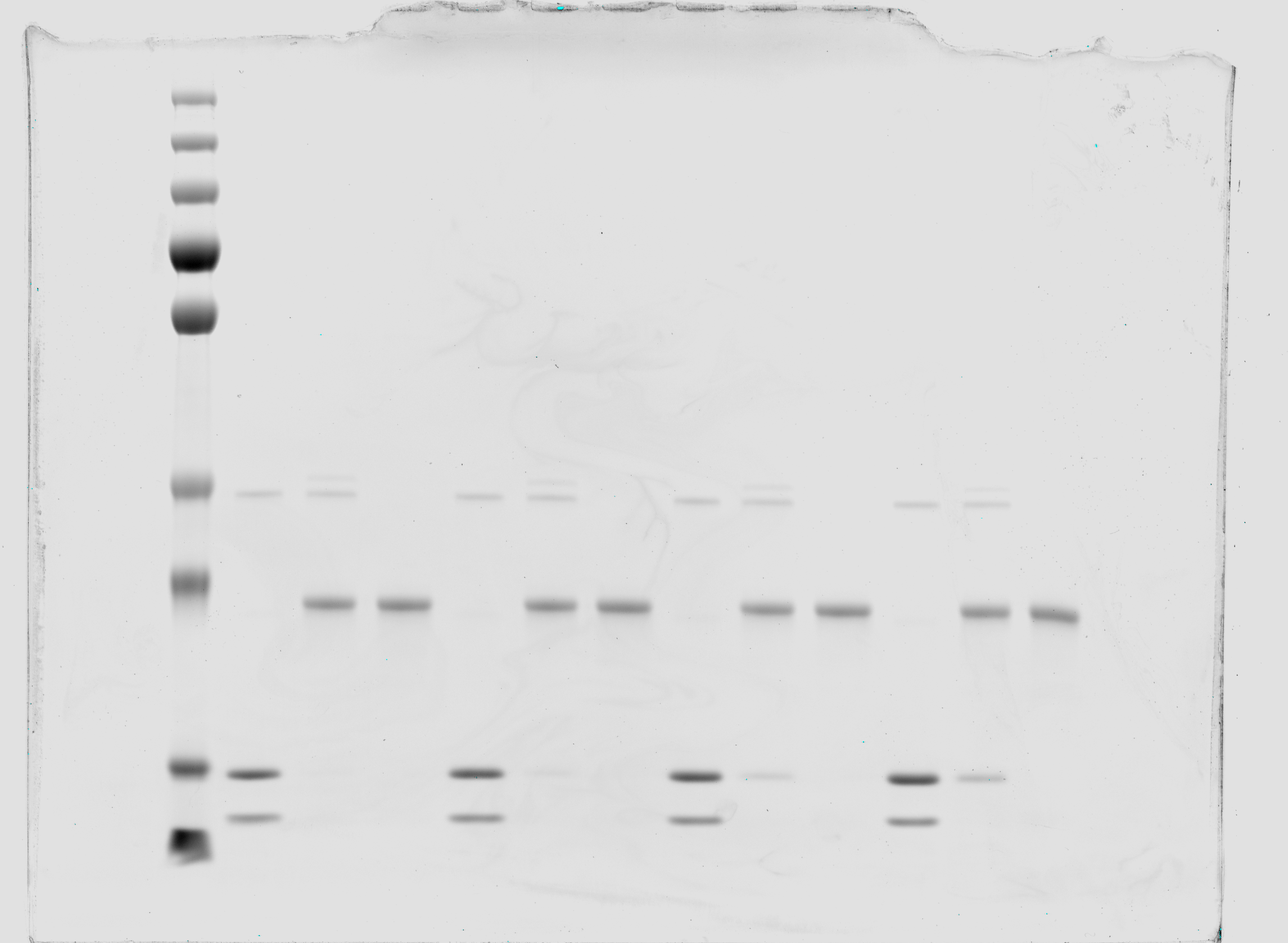

Supplement: Figure 6—source data 2. [file elife-102765-fig6-data2.zip › Figure 6A and 6B - Source Data.TIF.tif]

**A**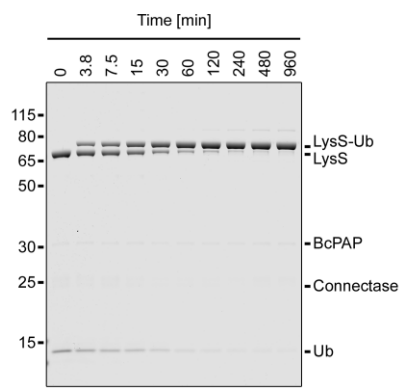**B**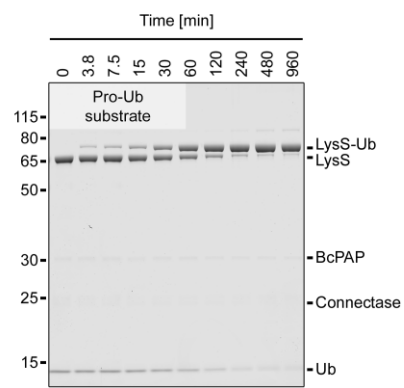

Supplement: Appendix 1—figure 2—source data 1. [file elife-102765-app1-fig2-data1.pdf]

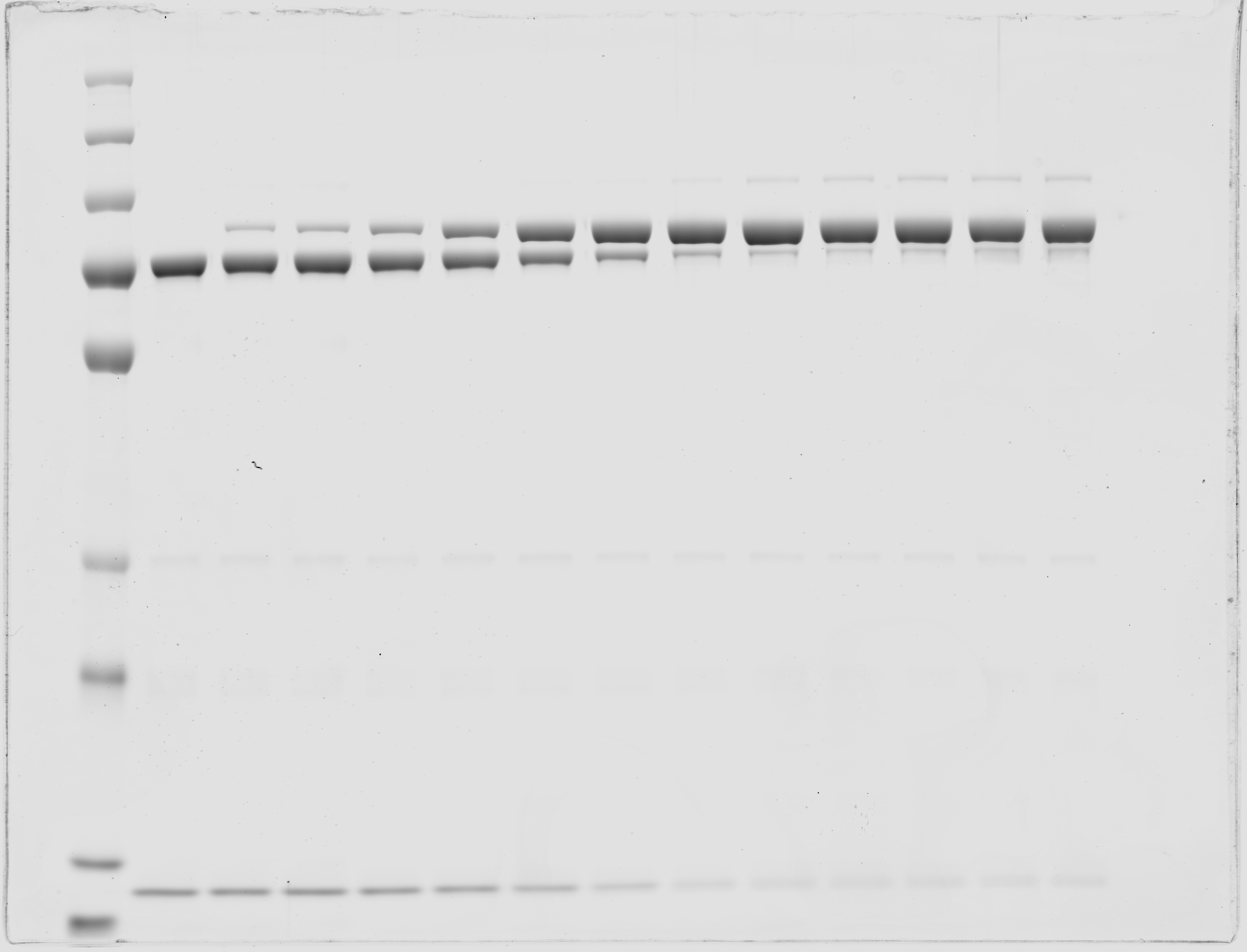

Supplement: Appendix 1—figure 2—source data 2. [file elife-102765-app1-fig2-data2.zip › Appendix 1- Figure 2B - Source Data.tif]

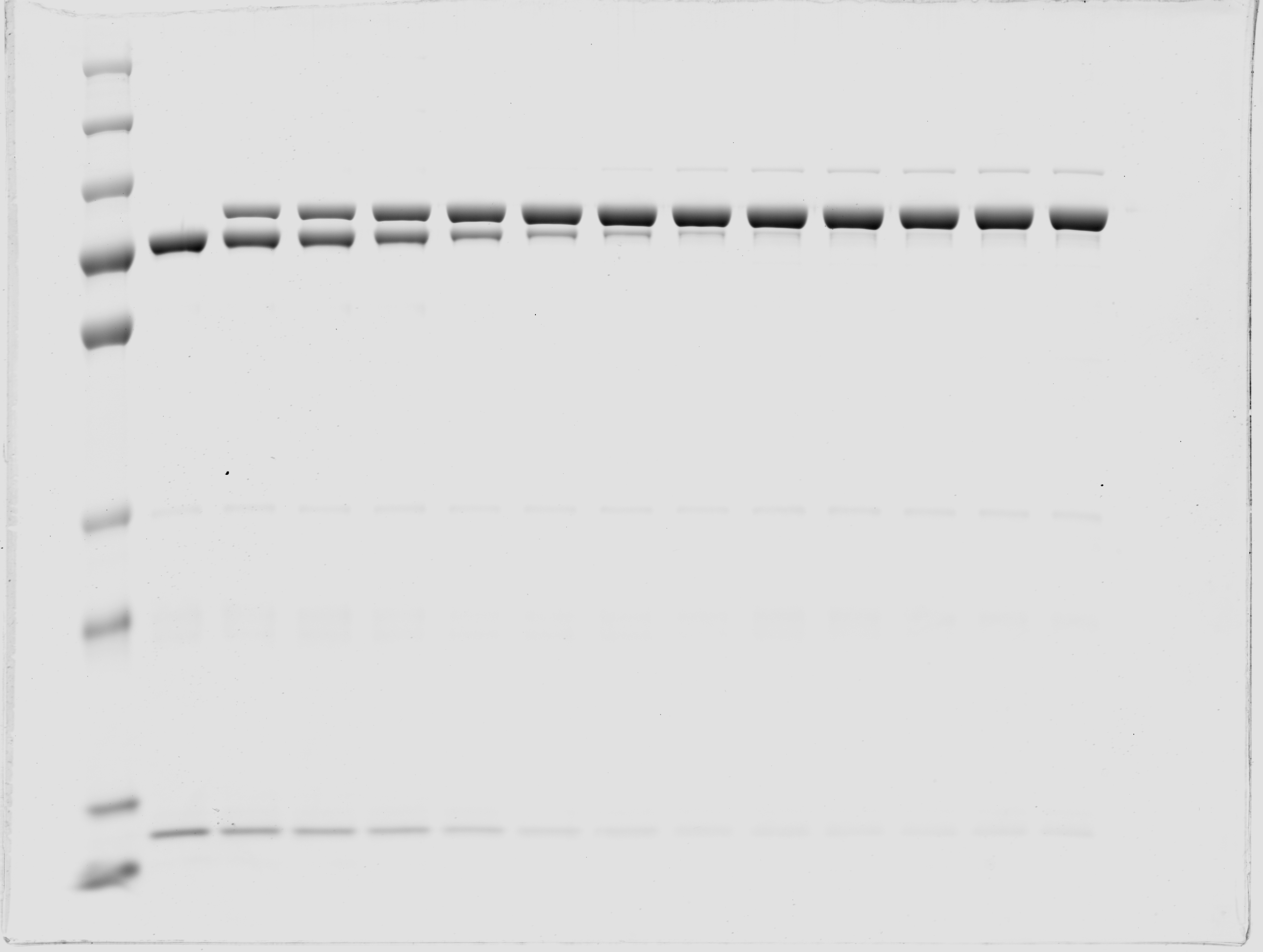

Supplement: Appendix 1—figure 2—source data 2. [file elife-102765-app1-fig2-data2.zip › Appendix 1- Figure 2A - Source Data.tif]

## Upper gel

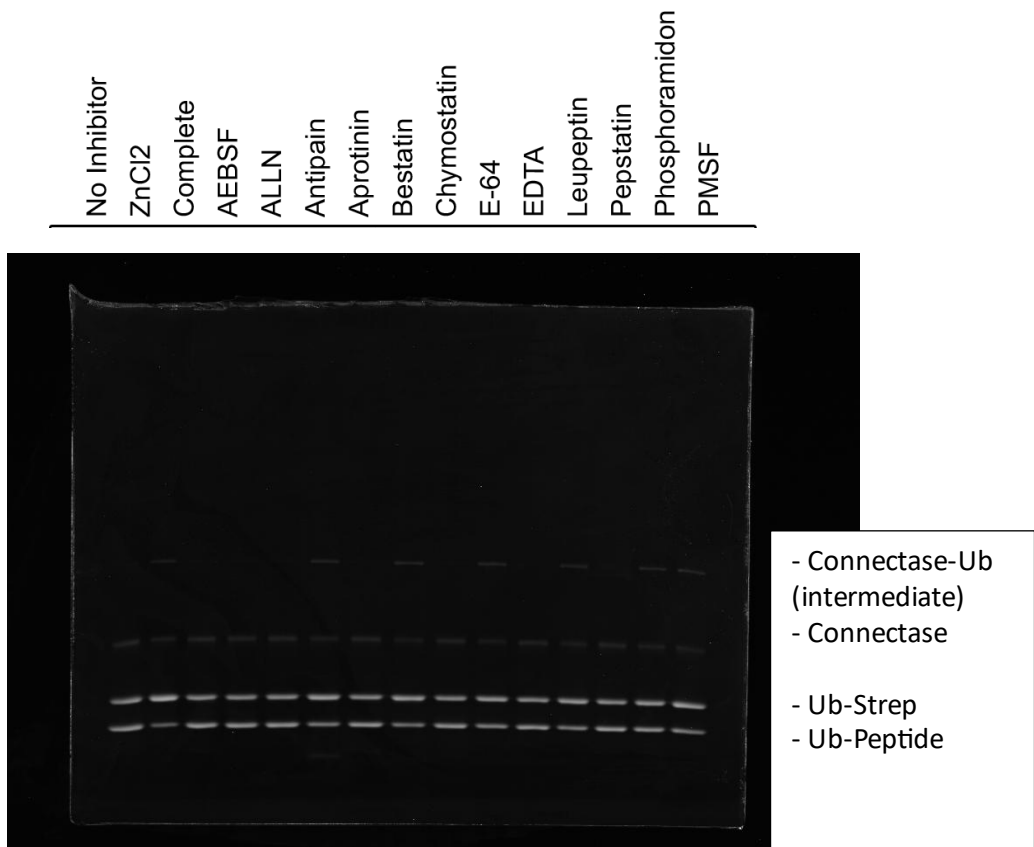

## Lower gel

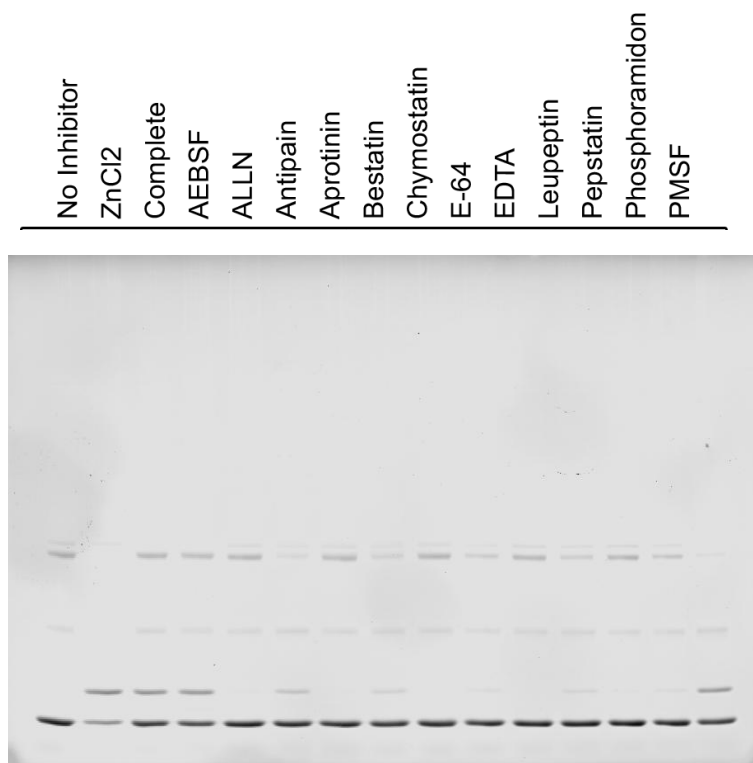

Supplement: Appendix 1—figure 4—source data 1. [file elife-102765-app1-fig4-data1.pdf]

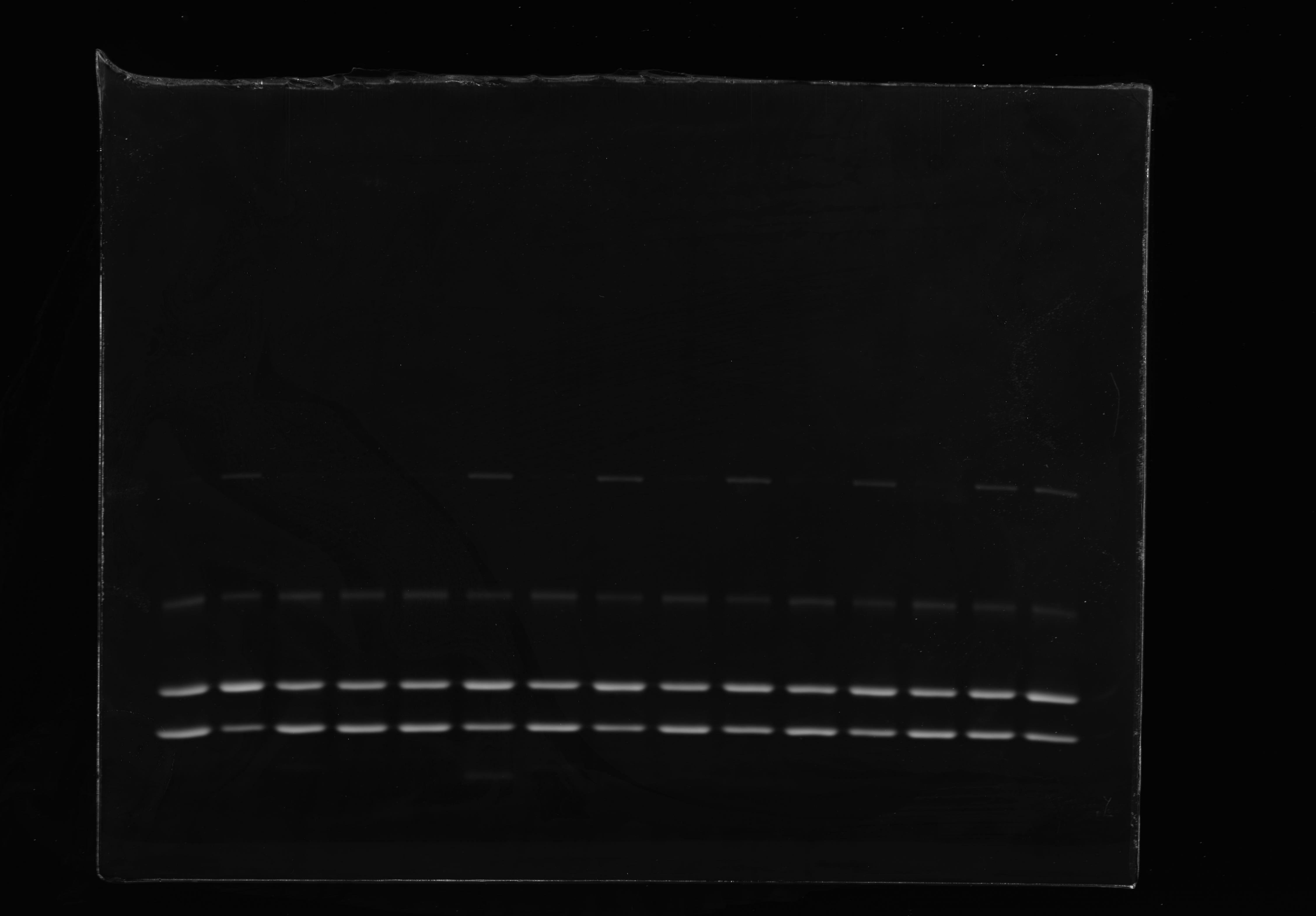

Supplement: Appendix 1—figure 4—source data 2. [file elife-102765-app1-fig4-data2.zip › Appendix 1 - Figure 4 - Source Data 1 - Connectase (upper gel).tif]

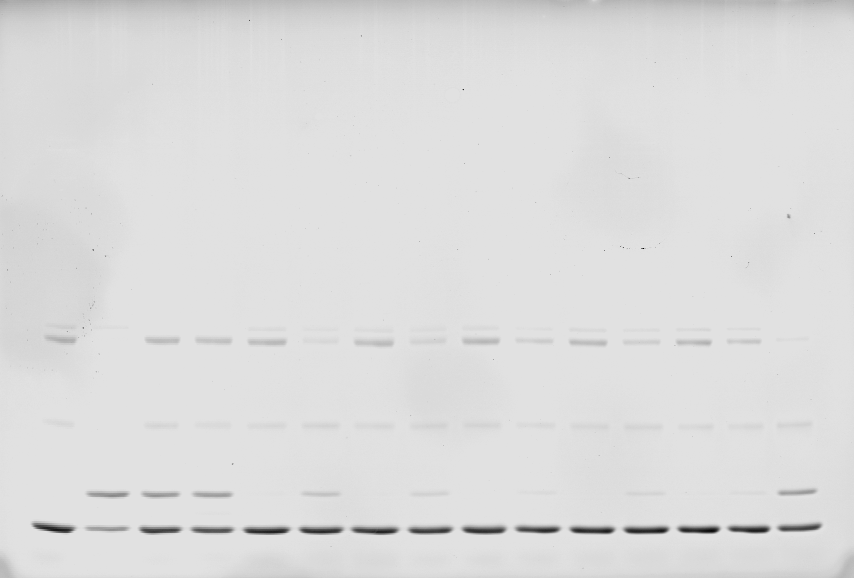

Supplement: Appendix 1—figure 4—source data 2. [file elife-102765-app1-fig4-data2.zip › Appendix 1 - Figure 4 - Source Data 2 -Connectase + BcPAP (lower gel).tif]

**A**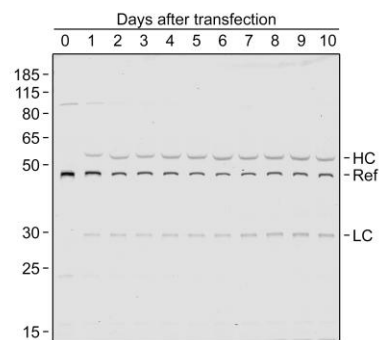**B**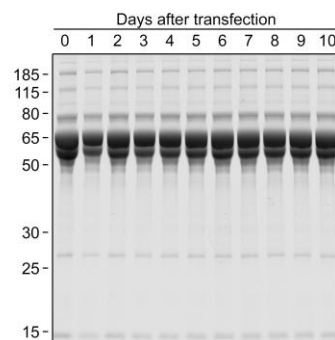

Supplement: Appendix 1—figure 5—source data 1. [file elife-102765-app1-fig5-data1.pdf]

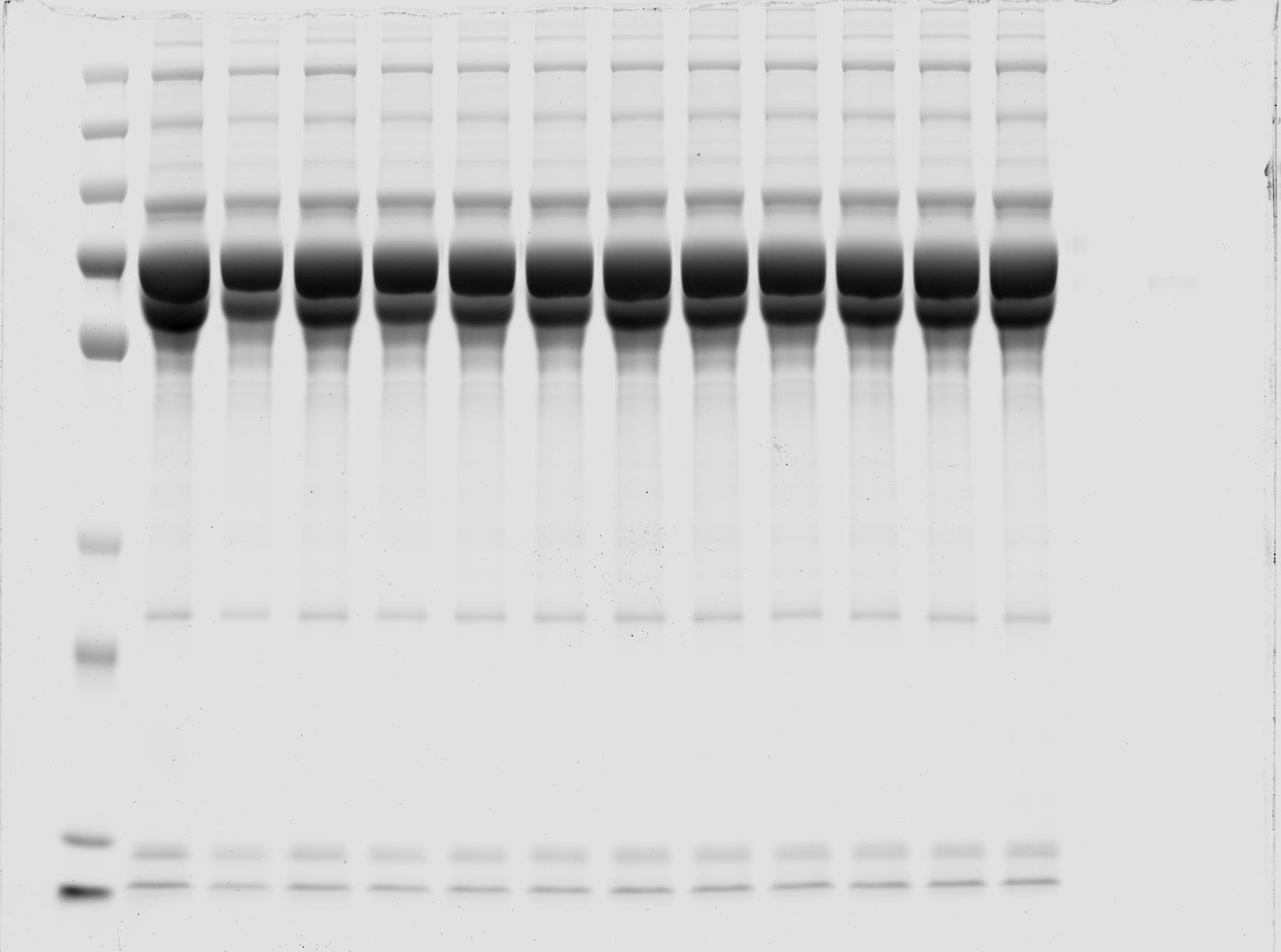

Supplement: Appendix 1—figure 5—source data 2. [file elife-102765-app1-fig5-data2.zip › Appendix 1 - Figure 5 - Source Data 1 -Coomassie stain.TIF.tif]

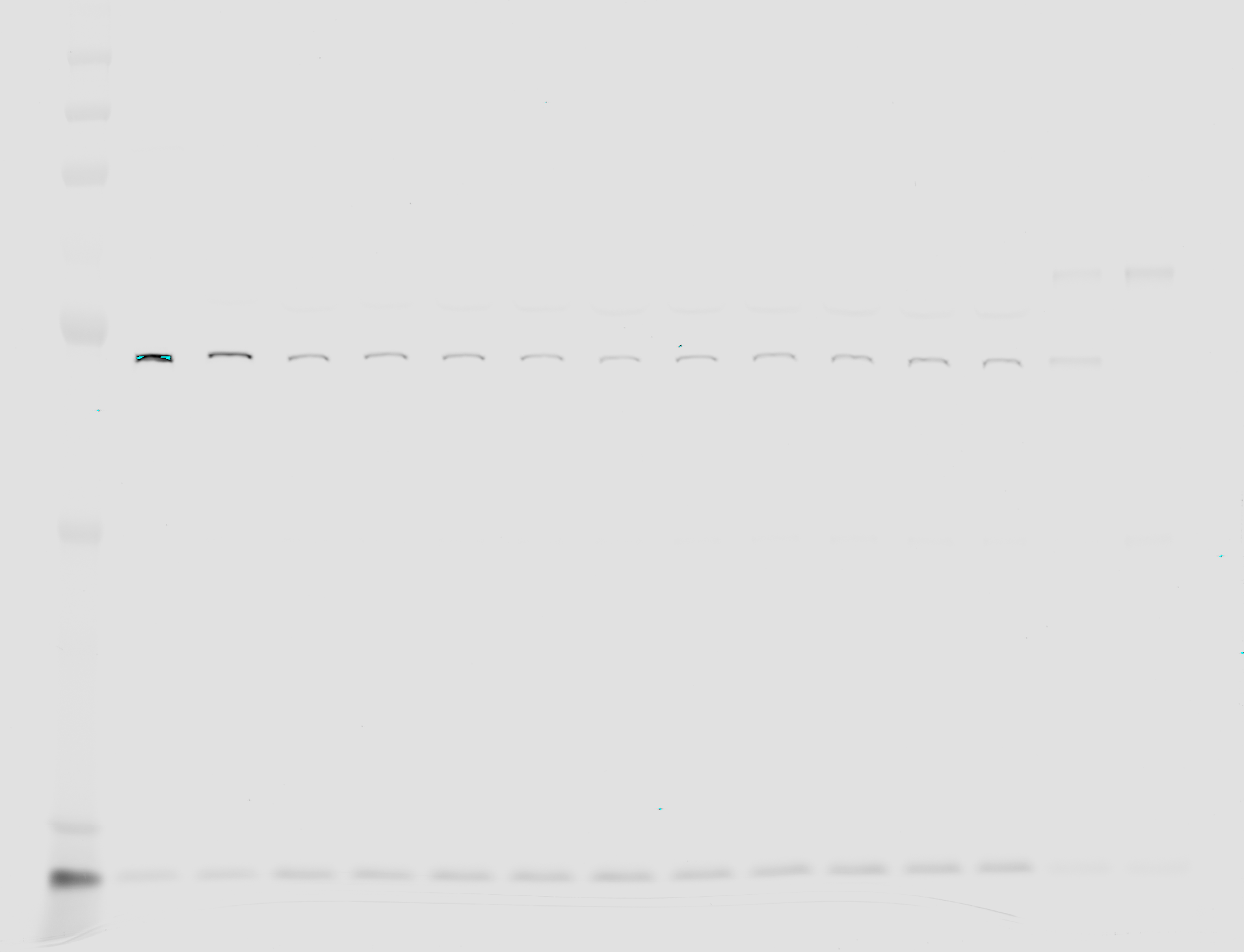

Supplement: Appendix 1—figure 5—source data 2. [file elife-102765-app1-fig5-data2.zip › Appendix 1 - Figure 5 - Source Data 2 -In-gel fluorescence.TIF.tif]

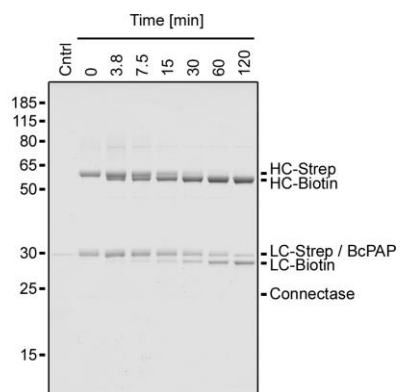

Supplement: Appendix 1—figure 6—source data 1. [file elife-102765-app1-fig6-data1.pdf]

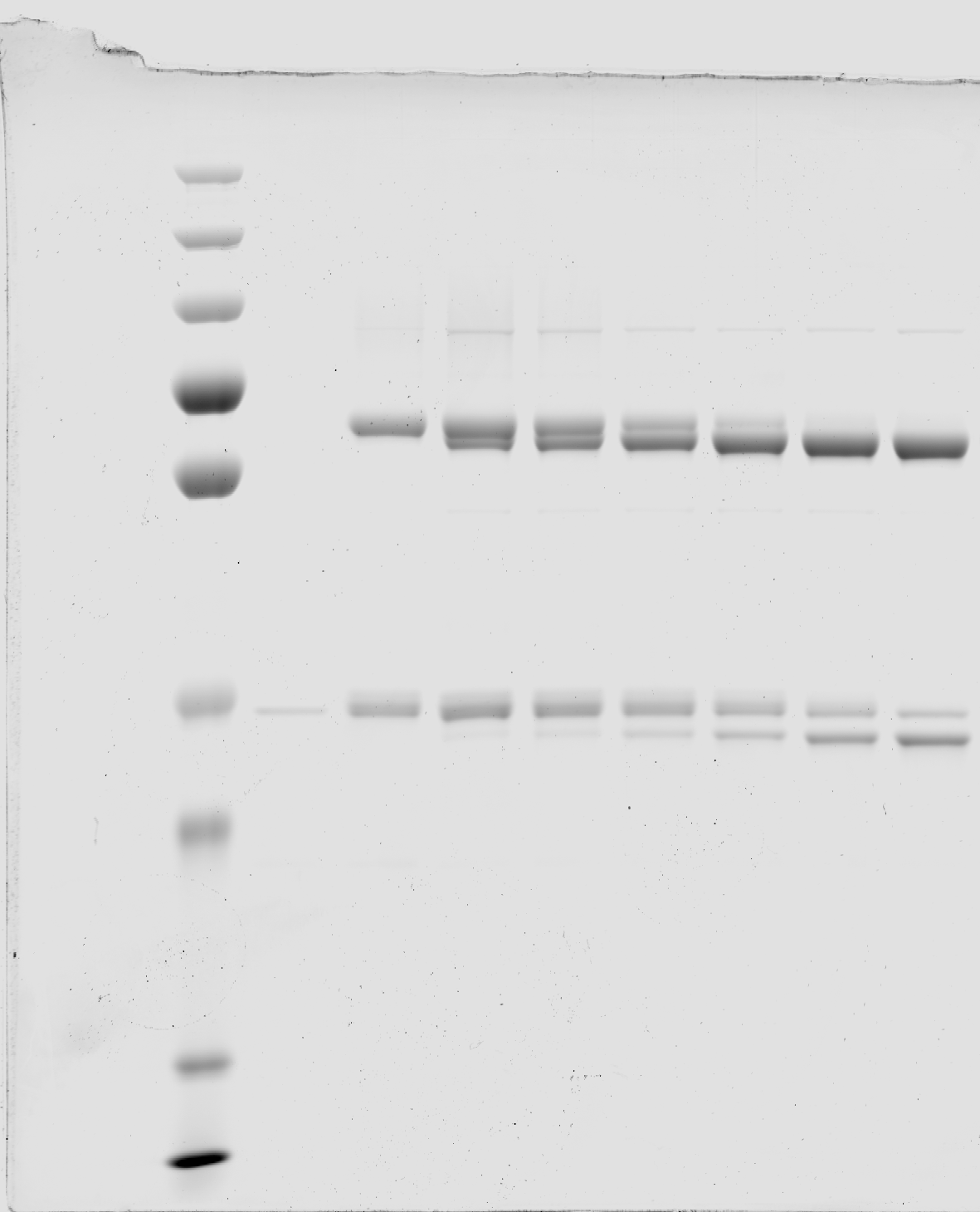

Supplement: Appendix 1—figure 6—source data 2. [file elife-102765-app1-fig6-data2.zip › Appendix 1 - Figure 6 - Source Data.TIF.tif]

**A**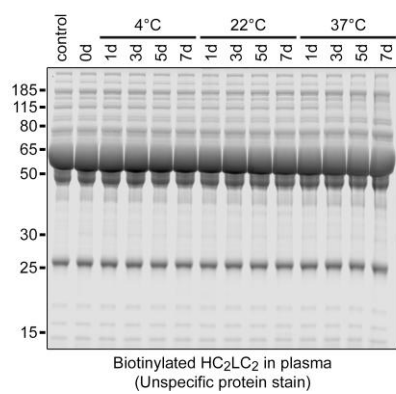**B**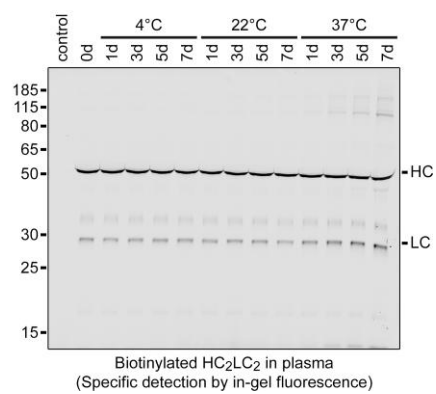

Supplement: Appendix 1—figure 7—source data 1. [file elife-102765-app1-fig7-data1.pdf]

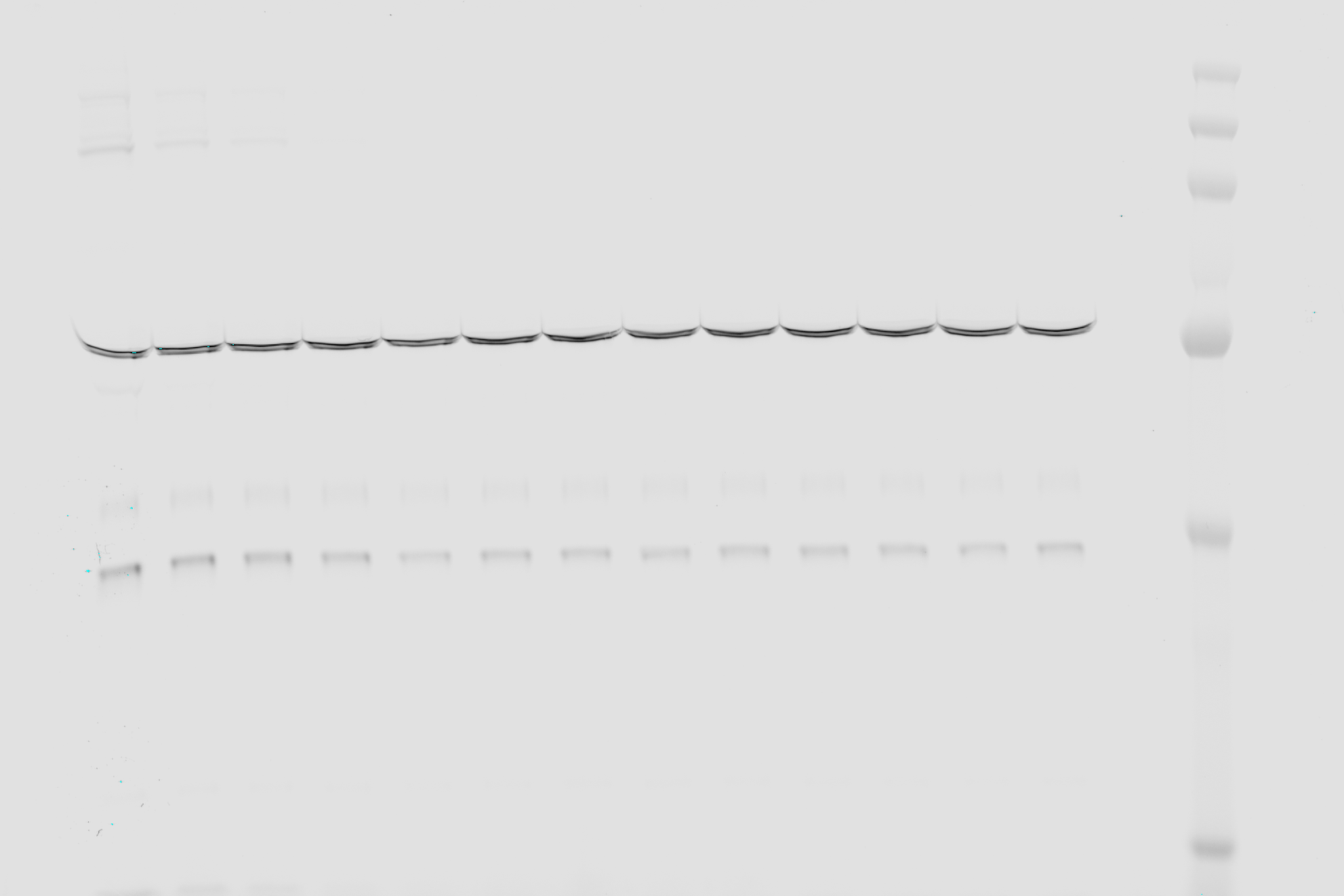

Supplement: Appendix 1—figure 7—source data 2. [file elife-102765-app1-fig7-data2.zip › Appendix 1 - Figure 7B - Source Data.TIF.tif]

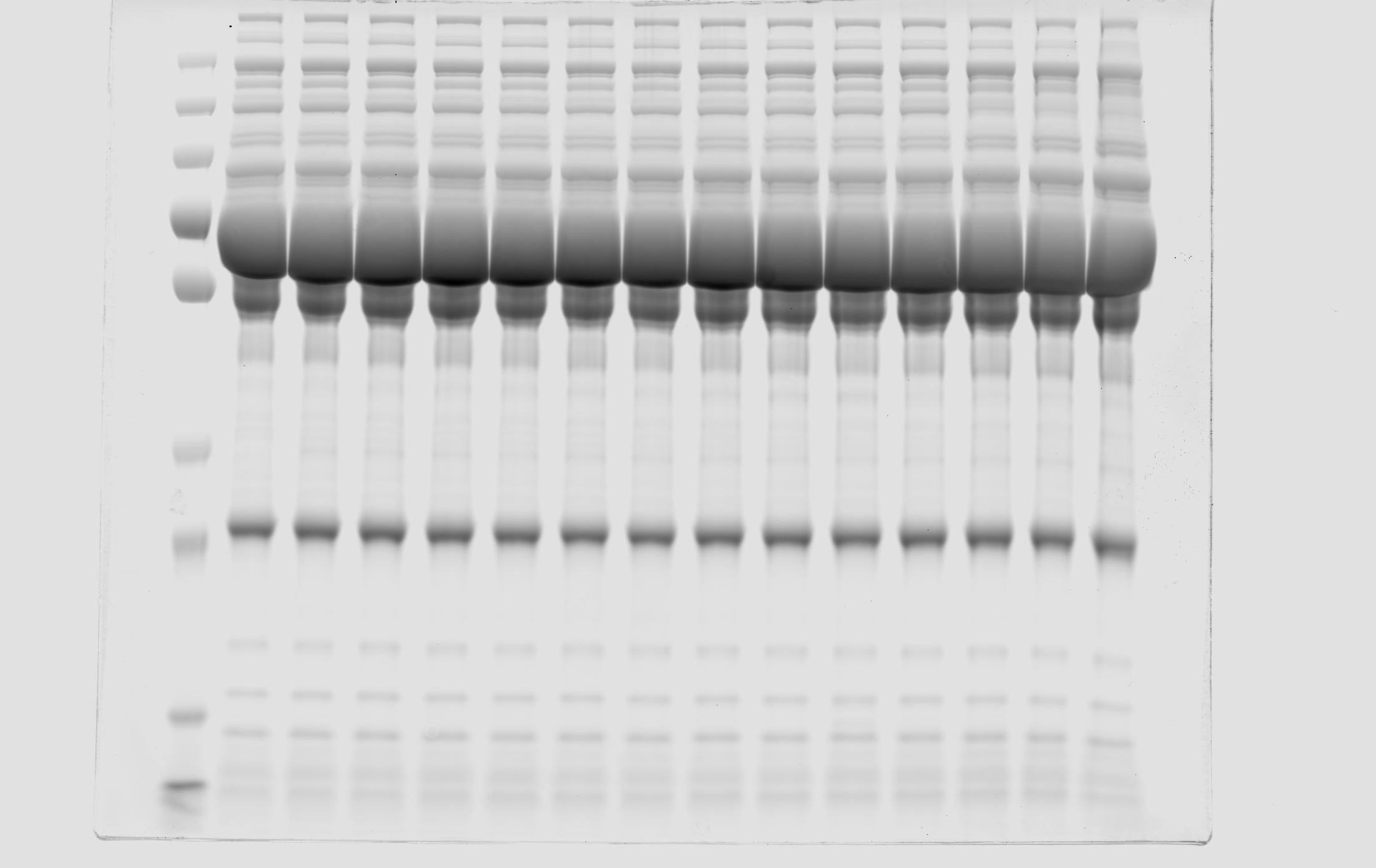

Supplement: Appendix 1—figure 7—source data 2. [file elife-102765-app1-fig7-data2.zip › Appendix 1 - Figure 7A - Source Data.tif.tif]

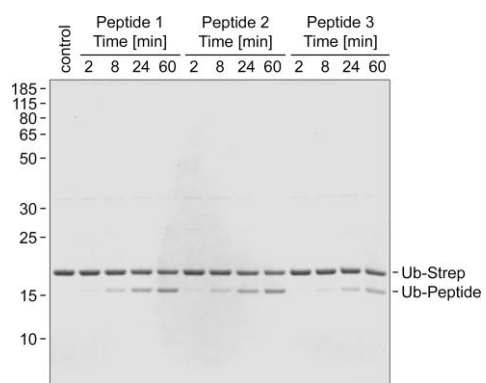

Supplement: Appendix 1—figure 8—source data 1. [file elife-102765-app1-fig8-data1.pdf]

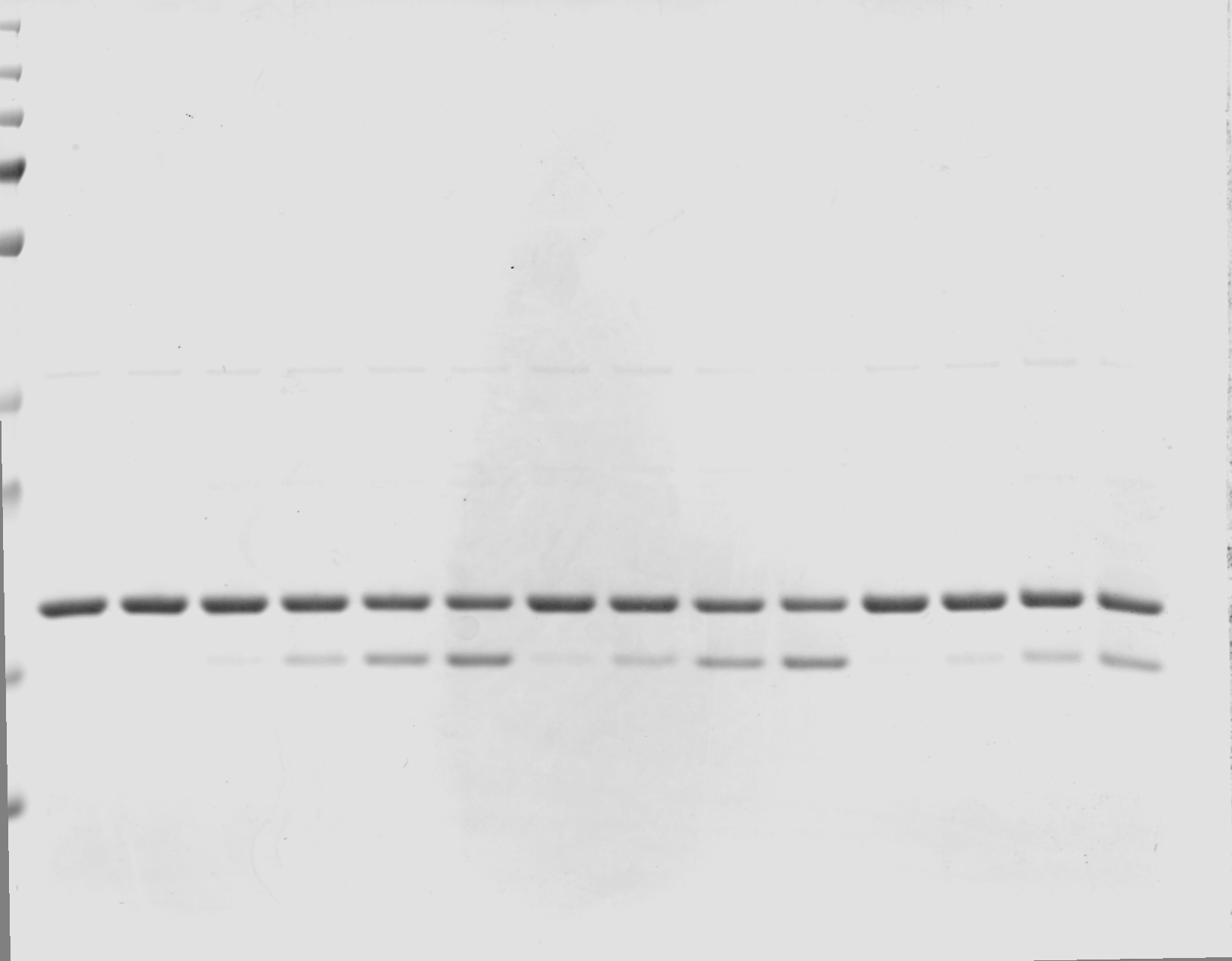

Supplement: Appendix 1—figure 8—source data 2. [file elife-102765-app1-fig8-data2.zip › Appendix 1 - Figure 8 - Source Data.tif]
